# Coactivator-independent vitamin D receptor signaling causes severe rickets in mice, that is not prevented by a diet high in calcium, phosphate, and lactose

**DOI:** 10.1038/s41413-024-00343-7

**Published:** 2024-08-20

**Authors:** Stefanie Doms, Lieve Verlinden, Iris Janssens, Justine Vanhevel, Roy Eerlings, René Houtman, Shigeaki Kato, Chantal Mathieu, Brigitte Decallonne, Geert Carmeliet, Annemieke Verstuyf

**Affiliations:** 1 Department of Chronic diseases and metabolism, Laboratory of Clinical and Experimental Endocrinology, KU Leuven, Leuven, Belgium; 2 Department of Cellular and Molecular Medicine, Laboratory of Molecular Endocrinology, KU Leuven, Leuven, Belgium; 3 Institute of Applied Microbiology, RWTH Aachen University, Aachen, Germany; 4 Precision Medicine Lab, Oss, The Netherlands; 5 Health Sciences Research Center, Iryo Sosei University, Iwaki, Fukuchima Japan; 6 Research Institute of Innovative Medicine, Tokiwa Foundation, Iwaki, Fukuchima Japan

**Keywords:** Calcium and vitamin D, Bone, Bone quality and biomechanics, Metabolic bone disease

## Abstract

The vitamin D receptor (VDR) plays a critical role in the regulation of mineral and bone homeostasis. Upon binding of 1α,25-dihydroxyvitamin D_3_ to the VDR, the activation function 2 (AF2) domain repositions and recruits coactivators for the assembly of the transcriptional machinery required for gene transcription. In contrast to coactivator-induced transcriptional activation, the functional effects of coactivator-independent VDR signaling remain unclear. In humans, mutations in the AF2 domain are associated with hereditary vitamin D-resistant rickets, a genetic disorder characterized by impaired bone mineralization and growth. In the present study, we used mice with a systemic or conditional deletion of the VDR-AF2 domain (*Vdr*^*ΔAF2*^) to study coactivator-independent VDR signaling. We confirm that ligand-induced transcriptional activation was disabled because the mutant VDR^ΔAF2^ protein was unable to interact with coactivators. Systemic *Vdr*^*ΔAF2*^ mice developed short, undermineralized bones with dysmorphic growth plates, a bone phenotype that was more pronounced than that of systemic *Vdr* knockout (*Vdr*^*−/−*^) mice. Interestingly, a rescue diet that is high in calcium, phosphate, and lactose, normalized this phenotype in *Vdr*^*−/−*^, but not in *Vdr*^*ΔAF2*^ mice. However, osteoblast- and osteoclast-specific *Vdr*^*ΔAF2*^ mice did not recapitulate this bone phenotype indicating coactivator-independent VDR effects are more important in other organs. In addition, RNA-sequencing analysis of duodenum and kidney revealed a decreased expression of VDR target genes in systemic *Vdr*^*ΔAF2*^ mice, which was not observed in *Vdr*^*−/−*^ mice. These genes could provide new insights in the compensatory (re)absorption of minerals that are crucial for bone homeostasis. In summary, coactivator-independent VDR effects contribute to mineral and bone homeostasis.

## Introduction

Active vitamin D, 1α,25-dihydroxyvitamin D_3_ [1,25(OH)_2_D_3_], is a key regulator of calcium and phosphate (re)absorption and is crucial for calcium, phosphate, and bone homeostasis. The vitamin D precursor, 25(OH)D_3_, is converted into 1,25(OH)_2_D_3_ by 1α-hydroxylase that is encoded by *Cyp27b1*. 1,25(OH)_2_D_3_ exerts its functions through binding to the vitamin D receptor (VDR; NR1I1), which regulates gene expression by acting as a ligand-induced transcription factor.^1^

Upon binding of 1,25(OH)_2_D_3_ to the ligand binding domain (LBD) of the VDR, the VDR undergoes conformational changes that evoke the association with the retinoid X receptor (RXR) and the repositioning of the carboxyterminal helix 12, which contains the activation function 2 (AF2) domain of the VDR. Within the nucleus, the VDR-RXR heterodimer binds to vitamin D responsive elements (VDRE) in the genome via two conserved zinc fingers in its amino terminal DNA-binding domain (DBD). Next, the 1,25(OH)_2_D_3_-bound VDR-RXR heterodimer initiates downstream gene-expression through coactivator recruitment to the repositioned AF2 domain, resulting in histone modifications, chromatin remodeling, and RNA polymerase II recruitment.^1–3^ Alternatively, the VDR is able to repress gene transcription via interaction of its LBD with corepressors. However, as X-ray crystal structures of corepressor peptides bound to VDR have not been reported, the exact location at which corepressors bind the VDR remains unclear, but is suggested to be independent of the AF2 domain. Interaction of the VDR with corepressors reduces its transcriptional activity by disrupting its interaction with transcription-promoting complexes and by tightening the chromatin structure.^4^

Interestingly, in mice and humans, inactivating mutations in *CYP27B1* and *VDR* result in hypocalcemia, hypophosphatemia, secondary hyperparathyroidism and short, deformed bones with dysmorphic growth plates. However, the mineral and skeletal phenotype of *Cyp27b1* knockout mice is more severe than that of *Vdr* knockout (*Vdr*^*−/−*^) mice and cannot be fully rescued by a diet high in calcium, phosphate, and lactose.^5–10^ In mice and humans, functional mutations in *CYP27B1* do not result in alopecia, while *VDR* mutations that disrupt its DNA binding do cause alopecia that is not reversed or prevented by a diet high in calcium, phosphate, and lactose. Thus, the presence of a functional VDR in absence of 1,25(OH)_2_D_3_ is sufficient to maintain hair follicle homeostasis.^11^ Indeed, the VDR can bind to VDREs in the genome in absence of 1,25(OH)_2_D_3_ and interact with corepressors, such as Hairless, described to inhibit the Wnt signaling pathway, which is crucial to maintain hair follicle homeostasis in keratinocytes.^12–15^ Such repressive signaling in the absence of ligand has been described for other nuclear receptors, including the thyroid hormone receptor (TR) and peroxisome proliferator-activated receptor (PPAR).^16–19^

Clearly, the VDR has the ability to repress important genes and pathways. However, continuous coactivator-dependent transcriptional induction by physiological levels of 1,25(OH)_2_D_3_ likely hinder the detection of targets that can also be repressed by the VDR. We hypothesize that the interaction of the VDR with corepressors is largely independent of its interaction with coactivators, and that we can reveal novel VDR targets by disrupting its interaction with coactivators. Therefore, in the present study, we used a transgenic mouse model expressing a VDR that lacks its AF2 domain (*Vdr*^*ΔAF2*^).^20^ We performed a detailed study of the bone phenotype of this mouse model and compared it to that of *Vdr*^*−/−*^ mice, which have lost both transcriptional induction and repression.

## Results

### No coactivator- but preserved corepressor interaction with VDR^ΔAF2^ after 1,25(OH)_2_D_3_ binding

**Fig. 1:**
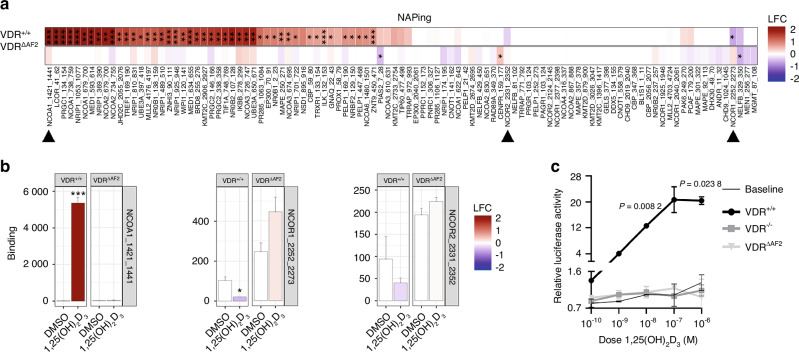
Increased corepressor but no coactivator interaction with VDR^ΔAF2^. **a** Log ten-fold change (LFC) difference in coregulator interactions between 1,25(OH)_2_D_3_- vs DMSO-treated VDR^+/+^ and VDR^ΔAF2^ proteins, measured by NAPing and ordered by hierarchical clustering (Euclidean distance, average linkage). **b** Absolute arbitrary unit of fluorescence as a measure of coregulator binding to the indicated (mutant) VDR, depicted for the indicated (black arrowheads) peptides of NCOA1, NCOR1, and NCOR2. Student’s t-test 1,25(OH)_2_D_3_ vs DMSO. Results are expressed as mean ± SD (4 technical replicates). **c** Luciferase transactivation assay to determine the 1,25(OH)_2_D_3_-induced transactivation capacity of the indicated (mutant) receptors, analyzed by two-way ANOVA followed by Dunnett multiple comparisons test of (mutant) receptor vs baseline. Results are expressed as mean ± SEM, (2 independent experiments with 3 technical replicates each)

To map interactions of the wild-type VDR (VDR^+/+^) and truncated VDR^ΔAF2^ proteins with coregulatory proteins, a chip platform for nuclear receptor activity profiling (NAPing) was used. The genomic mutation in *Vdr*^*−/−*^ mice resulted in a short VDR protein that was quickly degraded (Fig. S1). Therefore, this condition was not included in the NAPing assay. As expected, 1,25(OH)_2_D_3_ induced a shift in the coregulatory profile for VDR^+/+^, resulting in a significantly increased interaction with nuclear receptor coactivators (e.g. NCOAs), whereas interaction with nuclear receptor corepressors (e. g. NCOR1) was significantly downregulated (Fig. 1a, b). In contrast, 1,25(OH)_2_D_3_ did not induce coactivator binding to the VDR^ΔAF2^ protein, further proving the importance of the AF2 domain for coactivator binding. Interestingly, interaction of VDR^ΔAF2^ with the corepressor NCOR1 tended to be elevated in response to 1,25(OH)_2_D_3_ stimulation (Fig. 1a, b). Correspondingly, because of its inability to interact with coactivators, the VDR^ΔAF2^ protein was unable to transactivate a VDRE-containing reporter construct in an in vitro transfection assay (Fig. 1c). These data demonstrate that the VDR^ΔAF2^ protein is unable to activate gene transcription but suggest it can still exert repressive effects.

### Coactivator-independent VDR signaling negatively affects body weight and tibia length, which cannot be prevented by high dietary calcium, phosphate, and lactose supplementation

Previous studies in *Vdr*^*−/−*^ mice have demonstrated the detrimental effect of losing both VDR-mediated transcriptional induction and repression on mineral and bone homeostasis.^8–10,21^ In *Vdr*^*ΔAF2*^ mice on the other hand, the VDR^ΔAF2^ protein still interacts with corepressors, as shown by our NAPing assay, maintaining the possibility of transcriptionally repressing VDR target genes. To explore the phenotypical consequences of losing transcriptional induction but maintaining transcriptional repression, we compared 8-week-old *Vdr*^*ΔAF2*^ mice to 8-week-old *Vdr*^*−/−*^ mice on a normal diet (1% calcium, 0.7% phosphate) and on a rescue diet (2% calcium, 1.25% phosphate, 20% lactose).

**Fig. 2:**
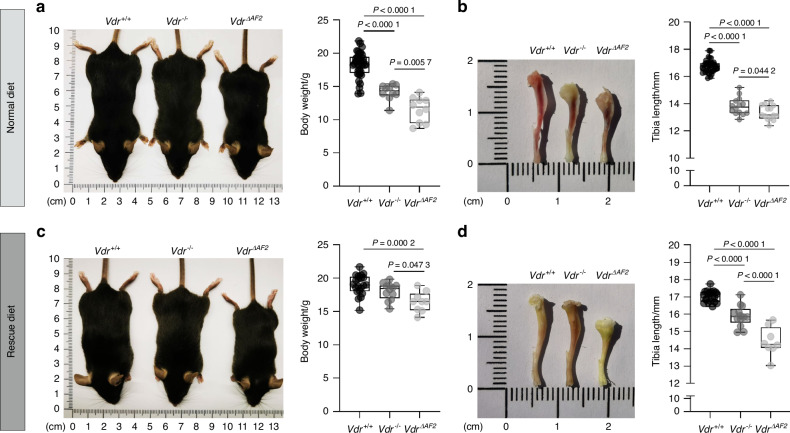
Phenotypic comparison of ligand-independent *Vdr*^ΔAF2^ and receptor-independent *Vdr*^*−/−*^ mice. Normal diet (upper panel) and rescue diet (bottom panel). **a,** **c** Representative pictures of mice and quantification of body weights and **b,** **d** representative pictures of tibiae and quantification of their lengths measured in 8-week-old female mice (*n* ≥ 8)

On the normal diet, body weight and tibia length of *Vdr*^*−/−*^ mice were significantly reduced compared to *Vdr*^*+/+*^ littermates (Fig. 2a, b). However, body weight and tibia length of *Vdr*^*ΔAF2*^ mice were even lower than that of *Vdr*^*−/−*^ mice (Fig. 2a, b). In addition, body weight and tibia length of *Vdr*^*−/−*^ were almost completely corrected by the rescue diet, whereas these parameters could not be normalized in *Vdr*^*ΔAF2*^ mice (Fig. 2c, d). Of note, unlike *Vdr*^*−/−*^ mice which started to develop alopecia from 8 weeks of age, *Vdr*^*ΔAF2*^ mice did not develop alopecia (Fig. S2). Clearly, phenotypic abnormalities were more pronounced in *Vdr*^*ΔAF2*^ mice compared to *Vdr*^*−/−*^ mice, suggesting that coactivator-independent VDR signaling/repression is even more deleterious than complete absence of genomic VDR signaling.

### Calcium, phosphate, and bone homeostasis are more severely impaired in *Vdr*^*ΔAF2*^ than in *Vdr*^*−/−*^ mice

**Fig. 3:**
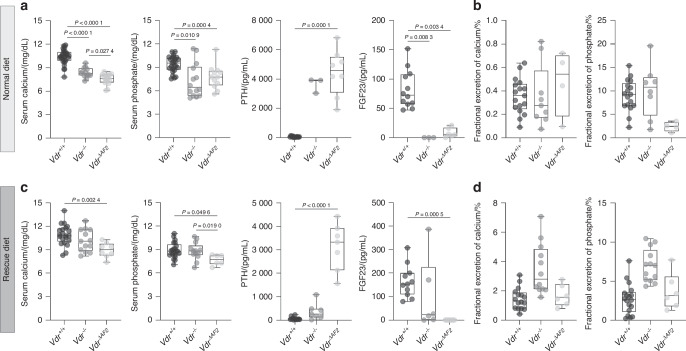
Calcium and phosphate homeostasis in *Vdr*^+/+^, *Vdr*^*−/−*^ and *Vdr*^ΔAF2^ mice. **a,** **c** Serum calcium, phosphate, PTH and FGF23 levels and **b, d** fractional excretion of calcium and phosphate /% of 8-week-old female mice weaned on a **a, b** normal diet or on a **c, d** rescue diet

On a normal diet, *Vdr*^*−/−*^ mice were hypocalcemic, hypophosphatemic, had elevated parathyroid hormone (PTH) levels and virtually undetectable fibroblast growth factor (FGF23) levels, while urinary fractional excretion of calcium and phosphate remained relatively high given the hypocalcemia and hypophosphatemia (Fig. 3a, b). *Vdr*^*ΔAF2*^ mice had similar serum phosphate, PTH, and FGF23 levels as observed in *Vdr*^*−/−*^ mice, whereas serum calcium was significantly lower compared to *Vdr*^*−/−*^ and *Vdr*^*+/+*^ mice (Fig. 3a, b). Interestingly, feeding a rescue diet normalized serum calcium, phosphate, PTH, and largely normalized FGF23 levels in *Vdr*^*−/−*^ mice (Fig. 3c), whereas fractional excretion of calcium and phosphate were slightly elevated (Fig. 3d). In contrast, *Vdr*^*ΔAF2*^ mice on this rescue diet remained hypocalcemic and hypophosphatemic and had highly elevated serum PTH and undetectable FGF23 levels, while fractional excretion of calcium and phosphate was similar to *Vdr*^*+/+*^ mice (Fig. 3c, d).

**Fig. 4:**
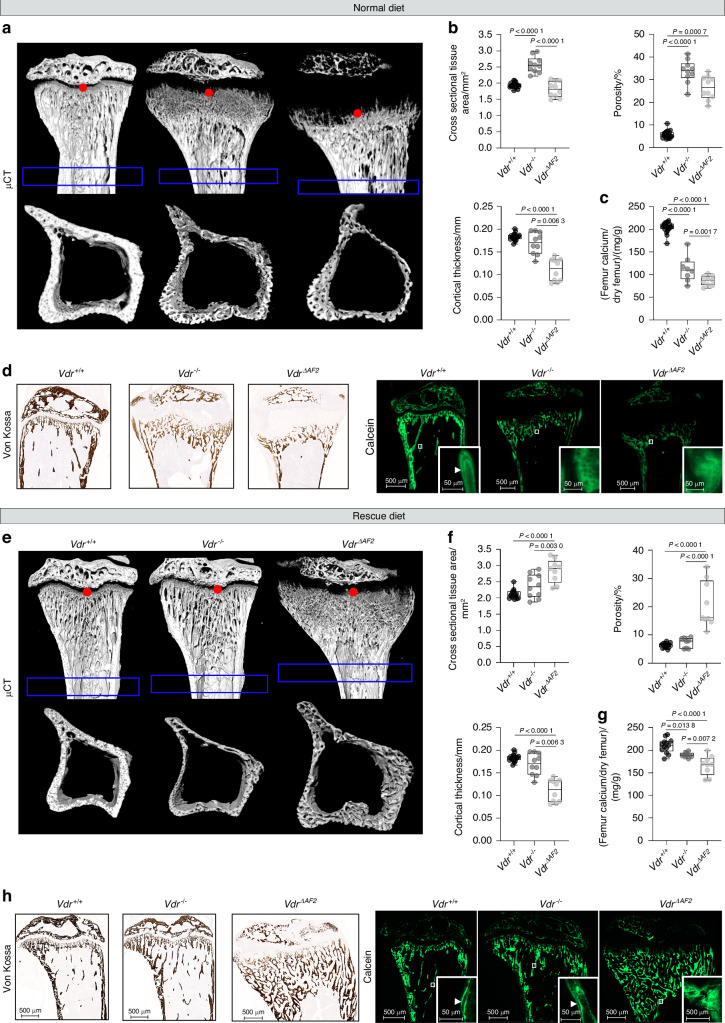
The rachitic bone phenotype of *Vdr*^ΔAF2^ mice is not prevented by a rescue diet high in calcium and phosphate. **a,** **e** Representative µCT images of the sagittal plane and cortical section of tibial bones, where red dots depict the reference point and blue boxes depict the analyzed cortical volume of interest. **b,** **f** Quantification of cortical bone parameters (cross-sectional tissue area, porosity, and cortical thickness). **c,** **g** Absolute calcium per femur dry weight. **d,** **h** Representative images of Von Kossa stained and calcein labeled –tibiae. All parameters are measured in 8-week-old, female *Vdr*^+/+^, *Vdr*^*−/−*^ and *Vdr*^ΔAF2^ mice on normal (upper panel) or rescue diet (lower panel)

We next evaluated the bone phenotype of *Vdr*^*−/−*^ and *Vdr*^*ΔAF2*^ mice by micro-computed tomography (µCT) analysis. When fed a normal diet, tibiae of *Vdr*^*−/−*^ mice showed extreme trabecularization of the metaphysial region, significantly increased cortical porosity and cross-sectional tissue area without changes in cortical thickness, compared to *Vdr*^*+/+*^ mice (Fig. 4a, b). The amount of calcium per femur dry weight, a measure of bone mineralization, was significantly decreased in *Vdr*^*−/−*^ mice compared to *Vdr*^*+/+*^ mice (Fig. 4c). Interestingly, on the normal diet, *Vdr*^*ΔAF2*^ mice had more pronounced metaphyseal trabecularization and significantly lower cortical thickness compared to *Vdr*^*−/−*^ mice (Fig. 4a, b). The amount of calcium per femur dry weight was also significantly lower in *Vdr*^*ΔAF2*^ mice than in *Vdr*^*−/−*^ mice (Fig. 4c). Von Kossa and calcein stainings visually confirmed that mineralization of the tibial bones was reduced in *Vdr*^*−/−*^ and especially in *Vdr*^*ΔAF2*^ mice compared to *Vdr*^*+/+*^ mice (Fig. 4d). The rescue diet largely prevented the bone phenotype of *Vdr*^*−/−*^ mice. Indeed, trabecularization of the metaphysical region was absent in *Vdr*^*−/−*^ mice on the rescue diet, and trabecular bone mass (data not shown) and cortical porosity were similar to that of *Vdr*^*+/+*^ mice (Fig. 4e, f). However, on this rescue diet cortical thickness remained significantly lower in *Vdr*^*−/−*^ mice than in *Vdr*^*+/+*^ littermates. Interestingly, although calcium and phosphate deposition in bone was markedly improved on the rescue diet (Fig. 4g, h), *Vdr*^*ΔAF2*^ mice persistently showed an aberrant bone phenotype including enlarged, undermineralized metaphysis and increased cortical porosity (Fig. 4e–h).

### Histological characterization of tibial bones confirms that bone homeostasis is more severely impaired in *Vdr*^*ΔAF2*^ than in *Vdr*^*−/−*^ mice

To better understand the differences in bone phenotype between *Vdr*^*ΔAF2*^ and *Vdr*^*−/−*^ mice, an extensive histological analysis was performed.

**Fig. 5:**
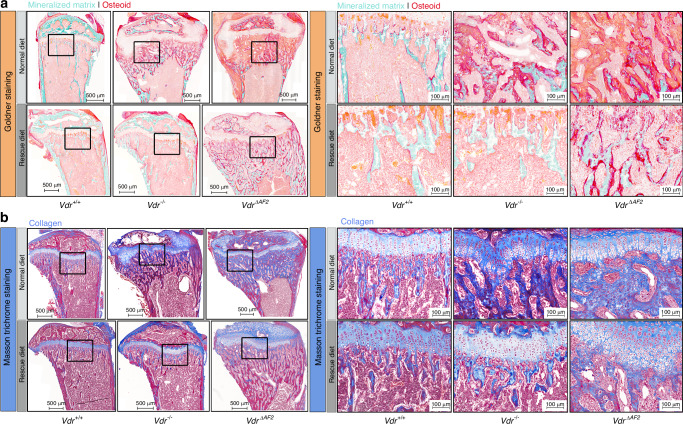
Histochemical comparison of *Vdr*^*+/+*^, *Vdr*^*−/−*^ and *Vdr*^*ΔAF2*^ mice; Goldner and Masson trichrome staining. Representative images showing overview (left panels) and metaphyseal regions (right panels) of **a** Goldner-stained sections depicting osteoid (red) deposition and **b** Masson trichrome-stained sections depicting collagen (blue) deposition in tibial sections of 8-week-old female *Vdr*^*+/+*^, *Vdr*^*−/−*^ and *Vdr*^*ΔAF2*^ mice on normal (upper panels) or rescue diet (lower panels). Left panel scale bars represent 500 µm, right panel scale bars represent 100 µm

**Fig. 6:**
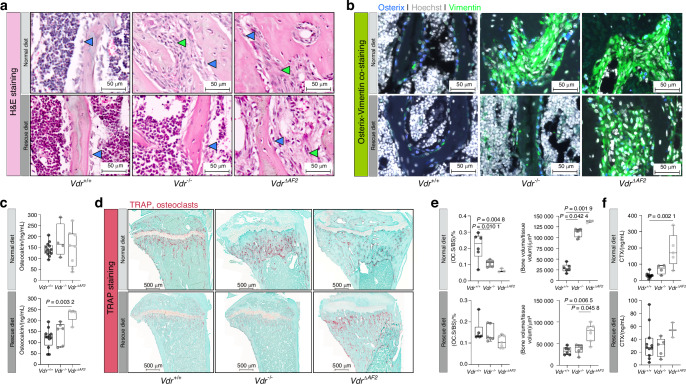
Histochemical and immunofluorescent comparison of *Vdr*^*+/+*^, *Vdr*^*−/−*^ and *Vdr*^*ΔAF2*^ mice; H&E, Osx-vimentin co-staining and TRAP staining. Representative images of metaphyseal regions on **a** H&E-stained sections (osteoblasts, and fibroblasts shaped cells are depicted by blue and green arrowheads, respectively) and **b** immunofluorescence using anti-Osx (blue) and anti-vimentin (green) antibodies. Cell nuclei are stained with Hoechst (blue). **c** Serum osteocalcin levels**. d** Representative overview images of TRAP stained sections and **e** quantification of (Oc.S/BS)/% and bone area/μm^2^ and **f** Serum CTX levels. All stainings and serum analyses were performed on samples from 8-week-old female *Vdr*^*+/+*^, *Vdr*^*−/−*^ and *Vdr*^*ΔAF2*^ mice on normal (upper panels) or rescue diet (lower panels). Scale bars represent 50 µm (a, b) or 500 µm (d)

Goldner staining revealed large areas of unmineralized bone matrix (osteoid) in *Vdr*^*−/−*^ and *Vdr*^*ΔAF2*^ mice on the normal diet. This excessive osteoid deposition was largely normalized by the rescue diet, although still present in *Vdr*^*ΔAF2*^ mice (Fig. 5a). Masson trichrome staining illustrated that the large unmineralized bone areas in *Vdr*^*−/−*^ and *Vdr*^*ΔAF2*^ mice on the normal diet and in *Vdr*^*ΔAF2*^ mice on the rescue diet mainly consisted of collagen (Fig. 5b). In addition, the Masson trichrome and Safranin-O staining clearly showed the growth plate abnormalities previously described in *Vdr*^*−/−*^ mice fed a normal diet. These growth plate abnormalities were persistent in *Vdr*^*ΔAF2*^ mice, but not in *Vdr*^*−/−*^ mice on the rescue diet (Fig. 5b, Fig. S3).^10,22^ Hematoxylin and eosin (H&E) staining revealed osteoblasts and fibroblast-shaped cells filling the bone marrow spaces between the undermineralized bone in *Vdr*^*−/−*^ and *Vdr*^*ΔAF2*^ mice, which persisted in the latter when given a rescue diet (Fig. 6a). Indeed, immunofluorescent labeling of osterix (Osx), an osteoblast marker, and vimentin, a mesenchymal cell marker highly expressed in fibroblasts, confirmed the presence of osteoblasts and fibroblasts, occupying the spaces between the undermineralized bone (Fig. 6b). In addition, serum osteocalcin levels, a marker for osteoblast activity, were increased in *Vdr*^*ΔAF2*^ mice on the rescue diet (Fig. 6c). On the other hand, total osteoclast surface over bone surface (OC.S/BS) tended to be decreased in *Vdr*^*−/−*^ and *Vdr*^*ΔAF2*^ mice on the normal diet, although, the (undermineralized) bone volume over tissue volume [(BV/TV)/%] was also significantly increased in both strains compared to *Vdr*^*+/+*^ littermates (Fig. 6d, e). On the rescue diet, no differences in OC.S/BS were observed, whereas BV/TV remained significantly increased in *Vdr*^*ΔAF2*^ mice compared to *Vdr*^*−/−*^ and *Vdr*^*+/+*^ mice (Fig. 6d, e). However, serum carboxy-terminal collagen crosslinks (CTx) levels, a marker for osteoclast activity, were only significantly increased in *Vdr*^*ΔAF2*^ mice on the normal diet but not in *Vdr*^*ΔAF2*^ mice on the rescue diet (Fig. 6f). Together, these findings suggest that coactivator-independent VDR signaling negatively affects mineral and bone homeostasis to a greater extent than absence of genomic VDR signaling, and that these effects cannot be completely prevented by feeding a diet high in calcium, phosphate, and lactose.

### No manifest role for coactivator-independent VDR signaling in bone cells

**Fig. 7:**
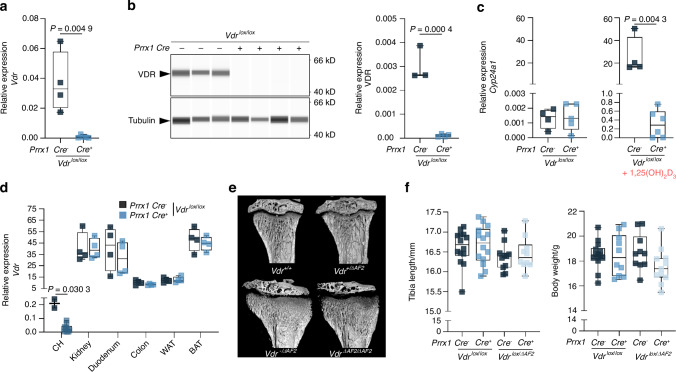
Validation of *Prrx1 Cre*-mediated *Vdr* knockdown. **a** qPCR and **b** simple western validation of *Vdr* gene and VDR protein expression in primary osetoblast cultures isolated from *Prrx1-Cre*^±^; *Vdr*^*lox/lox*^ mice (*n* = 3–5). **c** qPCR validation of *Cyp24a1* induction in primary osteoblasts, in basal conditions and after stimulation with 1,25(OH)_2_D_3_ (10^−8 ^mol/L). **d** qPCR validation of *Vdr* gene expression in chondrocytes (CH), kidney, duodenum, colon, white adipose tissue (WAT) and brown adipose tisse (BAT) isolated from *Prrx1-Cre*^±^*;Vdr*^*lox/lox*^ mice (*n* = 4). **e** representative µCT images of tibiae isoalated from *Vdr*^*+/+*^, *Vdr*^*+/*^^*-*^, *Vdr* ^*-/ΔAF2*^ mice (carrying one *Vdr* knockout allele and one *Vdr ΔAF2* allele) and *Vdr*^*ΔAF2/ΔAF2*^ (homozygous for the AF2 deletion). These mice were maintained on the recue diet in order to distinguish effects of the *Vdr* knockout allele from those of the *Vdr ΔAF2* allele **f** Tibia lengths and body weights measured in *Prrx1-Cre*^±^; *Vdr*^*lox/lox*^ and *Prrx1-Cre*^±^; *Vdr*^*lox/ΔAF2*^ mice (*n* = 10–15)

To differentiate direct actions of coactivator-independent VDR signaling within bone from its systemic effects on mineral supply to bone, we generated mice expressing the mutant VDR only in osteoblasts or osteoclasts by crossing paired related homeobox 1 (*Prrx1*)*-Cre* or M lysozyme (*LysM*)*-Cre* mice with *Vdr*^*lox/∆AF2*^ mice, respectively, and compared their phenotype with osteoblast- and osteoclast-specific *Vdr*^*−/−*^ mice, generated by crossing *Prrx1-Cre* or *LysM-Cre* mice with *Vdr*^*lox/lox*^ mice. We first validated the efficacy of the Cre-recombinase driven by the *Prrx1* promoter. In primary osteoblast cultures from *Prrx1-Cre*^+^ *;Vdr*^*lox/lox*^ mice*, Vdr* gene and VDR protein expression was significantly reduced (almost complete knockdown) compared to osteoblasts derived from *Prrx1-Cre-;Vdr*^*lox/lox*^ littermates (Fig. 7a, b). Treatment of these cells with 1,25(OH)_2_D_3_ induced the expression of *Cyp24a1*, a primary VDR target gene, although the level of induction was much more pronounced in *Prrx1-Cre*^*-*^*;Vdr*^*lox/lox*^ mice than in *Prrx1-Cre*^+^ *;Vdr*^*lox/lox*^ mice (Fig. 7c). *Prrx1-Cre* mediated deletion of *Vdr* expression was specific to osteochondrogenic lineage cells, as *Vdr* expression was, next to osteoblasts, significantly decreased in chondrocytes isolated from *Prrx1-Cre*^+^ *;Vdr*^*lox/lox*^ mice compared to those isolated from *Prrx1-Cre*^*-*^*;Vdr*^*lox/lox*^ mice. *Vdr* expression was unaltered in other *Vdr*-target tissues such as, kidney, duodenum, colon, white adipose tissue, and brown adipose tissue (Fig. 7d). To confirm that the introduction of only one *Vdr*^*ΔAF2*^ allele was sufficient to exert coactivator-independent effects, systemic *Vdr*^*-/ΔAF2*^ mice were compared to systemic *Vdr*^*+/ΔAF2*^ and *Vdr*^*ΔAF2/ΔAF2*^ mice. Both *Vdr*^*-/ΔAF2*^ and *Vdr*^*ΔAF2/ΔAF2*^ mice displayed the same rickets-like bone phenotype, whereas mice containing at least one *Vdr*^*+*^ allele had a normal bone phenotype (Fig. 7e). Body weights as well as tibia lengths were unaltered in both *Prrx1-Cre*^+^ *;Vdr*^*lox/lox*^ and *Prrx1-Cre*^+^ *;Vdr*^*lox/ΔAF2*^ mice compared to their *Cre-* littermates (Fig. 7f).

**Fig. 8:**
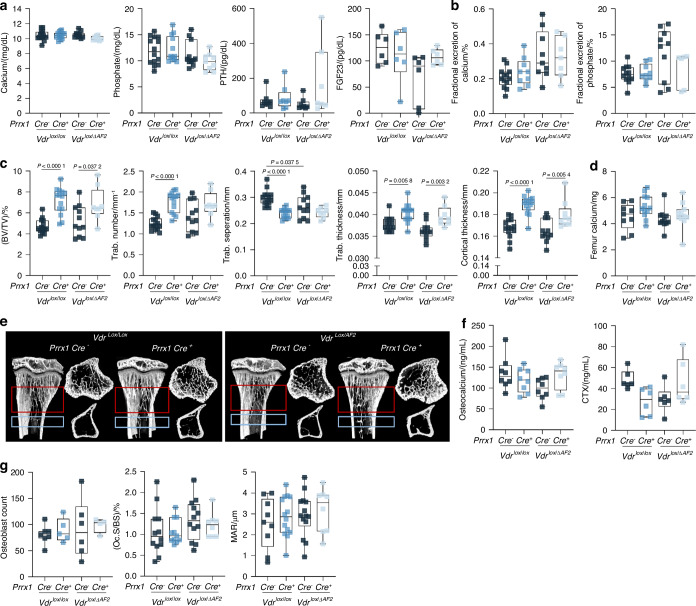
Increased bone mass in *Prrx1-Cre*^*+*^ *Vdr*^*lox/lox*^ and *Prrx1-Cre*^*+*^ *Vdr*^*lox/ΔAF2*^ mice versus their *Prrx1-Cre*^*-*^ littermates, without changes in calcium or phosphate homeostasis. **a** Serum calcium, phosphate, PTH, and FGF23 levels and **b** fractional excretion of calcium and phosphate (%). **c** Quantification of trabecular and cortical bone parameters; BV/TV (%), trabecular (trab.) number, separation, thickness, cortical thickness. **d** Absolute calcium content per femur. **e** Representative (based on average (BV/TV)/%) µCT images of the sagittal plane of the proximal tibia and the transverse plane of the indicated trabecular (red squares) and cortical (blue squares) selections. **f** Serum osteocalcin and CTX levels. **g** Quantification of osteoblast counts, OC.S/BS and mineral apposition rates (MAR), of which analyses were performed on two separate slides per tibia. All parameters were measured in 8-week-old female *Prrx1-Cre*^±^*;Vdr*^*lox/lox*^ and *Prrx1-Cre*^±^*;Vdr*^*lox/ΔAF2*^ mice (*n* = 6–14) weaned on normal diet (1% calcium, 0.7% phosphate)

Serum calcium, phosphate, PTH, and FGF23 levels were unaltered in both *Prrx1-Cre*^+^ *;Vdr*^*lox/lox*^ and *Prrx1-Cre*^+^ *;Vdr*^*lox/ΔAF2*^ mice compared to their *Cre-* littermates (Fig. 8a). Fractional excretion of calcium and phosphate was also normal (Fig. 8b). However, trabecular and cortical bone mass, assessed by µCT analysis, was similarly increased in *Prrx1-Cre*^+^ *;Vdr*^*lox/lox*^ and *Prrx1-Cre*^+^ *;Vdr*^*lox/ΔAF2*^ mice compared to their *Cre*^*-*^ littermates, as evidenced by increased BV/TV, increased trabecular number and thickness, and increased cortical thickness (Fig. 8c, e). Although trabecular and cortical bone mass was increased, bone calcium content measured in ashed femurs remained unaltered, as well as serum osteocalcin and CTx levels (Fig. 8f). Osteoblast numbers, assessed on H&E staining, OC.S/BS, assessed on tartrate resistant acid phosphatase (TRAP) staining, and the mineral opposition rate (MAR), assessed by analysis of calcein labels were similar in both strains (Fig. 8g). Finally, gene expression levels measured in femurs of *Prrx1-Cre;Vdr*^*lox/lox*^ and *Prrx1-Cre;Vdr*^*lox/ΔAF2*^ mice also remained largely unaltered (Fig. S4).

**Fig. 9:**
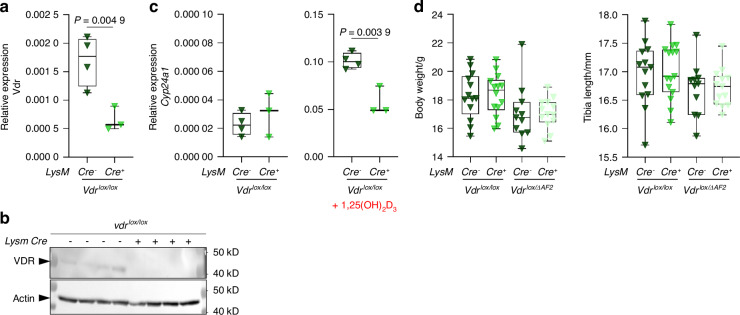
Validation of *LysM Cre* mediated *Vdr* knockdown. **a** qPCR and **b** western blot validation (not accurately quantifiable) of *Vdr* gene and VDR protein expression in primary osteoclast cultures, derived from hematopoetic cells, isolated from *LysM-Cre*^±^*;Vdr*^*lox/lox*^ mice (*n* = 3–4). **c** *Cyp24a1* transcript levels, assessed by qPCR analysis, in primary osteoclasts in basal conditions and after stimulation with 1,25(OH)_2_D_3_ (10^−8 ^mol/L). **d** Body weight and tibia length measured in *LysM-Cre* ^±^*;Vdr*^*lox/lox*^ and *LysM-Cre* ^±^*;Vdr*^*lox/ΔAF2*^ mice (*n* = 11–15)

In primary osteoclast cultures, initiated from hematopoietic cells derived from *LysM-Cre*^+^*;Vdr*^*lox/lox*^ mice, *Vdr* gene and VDR protein expression were significantly reduced (approximately 62%), compared to cultures from *LysM-Cre*^*-*^*;Vdr*^*lox/lox*^ littermates (Fig. 9a, b). *Cyp24a1* expression was also induced by 1,25(OH)_2_D_3_ and induction was more pronounced in cells isolated from *Cre-;Vdr*^*lox/lox*^ mice than in cells isolated from *Cre*^+^;*Vdr*^*lox/lox*^ mice (Fig. 9c). However, we did not observe any phenotypic differences between *LysM-Cre*^+^*; Vdr*^*lox/lox*^_*/*_*Vdr*^*lox/ΔAF2*^ and *LysM*-*Cre*^*-*^*; Vdr*^*lox/lox*^_*/*_*Vdr*^*lox/ΔAF2*^ mice, nor between *LysM-Cre* ^±^*; Vdr*^*lox/lox*^ and *LysM-Cre* ^±^*; Vdr*^*lox/ΔAF2*^ mice (Fig. S5).

These findings refute the idea that coactivator-independent signaling of the VDR has important direct effects in skeletal cells themselves and therefore suggest a more important role in intestine and kidney.

### Transcriptomic analysis suggests promising target genes of VDR-mediated repression involved in mineral ion transport

**Fig. 10:**
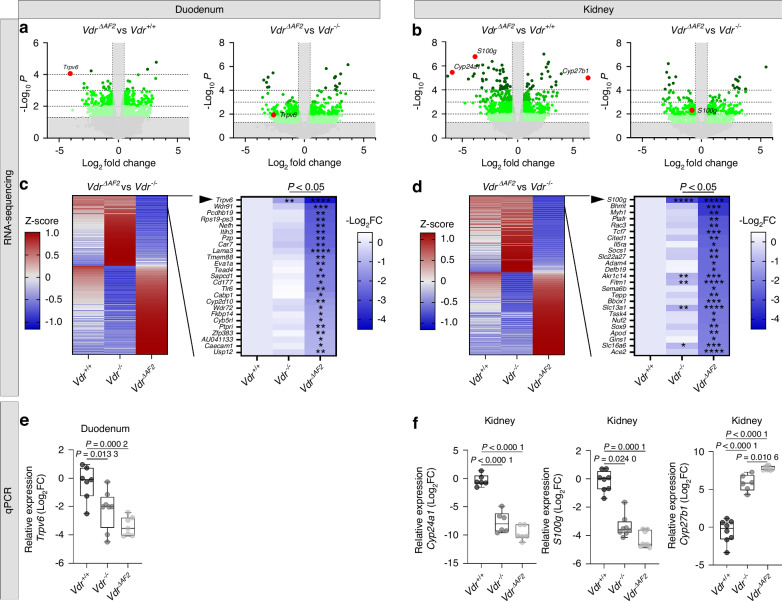
Transcriptomic analysis of duodenum and kidney of *Vdr*^*+/+*^, *Vdr*^*−/−*^ and *Vdr*^*ΔAF2*^ mice. **a,** **b** Volcano plots visualizing the differentially expressed genes in *Vdr*^*ΔAF2*^ vs *Vdr*^*+/+*^ and *Vdr*^*ΔAF2*^ vs *Vdr*^*−/−*^ for duodenum **a** and kidney **b.** Vertical and horizontal gray areas represent excluded data with log two-fold changes between −1.4 and 1.4 and *P* < 0.05, respectively. Well-known VDR target genes, *Trpv6*, *S100g*, *Cyp24a1* and *Cyp27b1* are indicated with red data points. (**c,d**, left panels) Heatmaps (z-score) visualizing all significantly (*P* < 0.05) differentially expressed genes between *Vdr*^*ΔAF2*^ and *Vdr*^*−/−*^ ordered by log two-fold change (Log_2_FC) difference between *Vdr*^*ΔAF2*^ and *Vdr*^*+/+*^. Of these, (**c,d**, right panels) top 25 genes that were downregulated in *Vdr*^*ΔAF2*^ are listed and visualized using heatmaps, ordered by log_2_FC difference between *Vdr*^*ΔAF2*^ and *Vdr*^*+/+*^. For these 25 genes, an additional Students’ *t*-test was performed to evaluate the difference between *Vdr*^*ΔAF2*^ or *Vdr*^*−/−*^ vs. *Vdr*^*+*/+^, *P < 0.05^, *^*P < 0.01^, *^**P < 0.001, ****P < 0.000 1. **e, f** qPCR validation of the indicated, known duodenal and renal VDR target genes, expressed as log_2_FC of relative gene expression (target gene/housekeeping gene) over average *Vdr*^+/+^ ( = 0)

To investigate differences in transcriptional regulation that could (at least in part) account for the more severe phenotype of *Vdr*^*ΔAF2*^ mice compared to *Vdr*^*−/−*^ mice, RNA-sequencing (RNA-seq) studies on duodenum and kidney of *Vdr*^+/+^, *Vdr*^*−/−*^ and *Vdr*^*ΔAF2*^ mice were performed. In duodenum, the expression of 1 389 genes was significantly (*P* < 0.05) changed (702 up; 687 down) when comparing *Vdr*^*ΔAF2*^ to *Vdr*^+/+^ mice. When comparing *Vdr*^*ΔAF2*^ to *Vdr*^*−/−*^ mice, 1 329 genes were differentially regulated (743 up; 586 down) (Fig. 10a). Similar numbers of differentially expressed genes were observed in kidney when comparing *Vdr*^*ΔAF2*^ to *Vdr*^+/+^ mice (881 up; 939 down) or to *Vdr*^*−/−*^ mice (693 up; 647 down) (Fig. 10b). To evaluate the difference between the complete loss of genomic VDR signaling and the loss of coactivator-dependent signaling, we further focused our analysis on the comparison between *Vdr*^*−/−*^ and *Vdr*^*ΔAF2*^ mice. Of the resulting list of significantly differentially regulated genes (Fig. 10c, d left panels), we selected the 25 most downregulated genes (Fig. 10c, d right panels). On top of this gene list, we found *Trpv6* (in duodenum) and *S100g* (in kidney), which are primary VDR target genes and important regulators of calcium homeostasis (Fig. 10c, d). Other genes in this list include genes involved in mineral ion transport (*Trpv6*, *Car7*, *Cabp1* and *Wdr72* in duodenum and *S100g*, *Slc22a27* and *Slc13a1* in kidney) and in fatty acid transport (*Bbox1* in kidney). To validate these RNA-seq results (Fig. 10a, b), well known VDR target genes [duodenum (*Trpv6*) and kidney (*Cyp24a1, S100g, Cyp27b1*)] were selected and verified by quantitative polymerase chain reaction (qPCR) (Fig. 10e, f).

## Discussion

VDR expression in intestine and kidney is crucial to maintain an adequate mineral supply to serum and to bone. However, some important VDR target genes and pathways involved in mineral transport may remain undetected due to the continuous coactivator-dependent transcriptional induction. Therefore, we used a transgenic mouse model expressing a VDR that lacks its AF2 domain (*Vdr*^*ΔAF2*^).^20^ The NAPing assay, performed on the full-length VDR^+/+^ and VDR^ΔAF2^ protein, confirmed that the VDR^ΔAF2^ was completely unresponsive to ligand-induced interaction with coactivators. This demonstrates that coactivator binding to the VDR is completely dependent on a functional AF2 domain. In contrast, co-repressor interaction with the VDR is AF2 domain-independent.^23^ This observation suggests that the VDR^ΔAF2^ may function as a transcriptional repressor, a function that has been described for unliganded nuclear receptors, such as TR and PPAR.^16,19^ However, ligand-independent repression is most likely not identical to AF2-independent repression, as some VDR target genes are repressed in a ligand-dependent manner.^24^ The *Vdr*^*ΔAF2*^ mouse model enabled us to investigate the effects of coactivator-independent but corepressor-dependent, repressive VDR signaling. As expected, *Vdr*^*ΔAF2*^ mice do not develop alopecia, as only mutations resulting in DNA binding defects, RXR dimerization defects, or complete absence of VDR cause these disturbances in hair cycling.^10,25–27^ Here, we show that the effects of coactivator-independent VDR signaling extend beyond those observed in hair follicle homeostasis,^14,28^ evidenced by the more severely impaired mineral homeostasis and bone phenotype observed in *Vdr*^*ΔAF2*^ mice compared to *Vdr*^*−/−*^ mice. We hypothesized that VDR^ΔAF2^-mediated repression has direct negative effects on skeletal cell function and/or inhibits mineral absorption.

We next targeted *Vdr* expression in osteoblast lineage cells by means of *Prrx1*-promoter driven recombination, which reduces both osteoblastic and chondrocytic *Vdr* expression. However, earlier studies demonstrated that chondrocytic *Vdr* expression has no impact on bone or mineral homeostasis in 8-week-old mice.^29^ Hence, the observed phenotype in *Prrx1-Cre*^+^ *;Vdr*^*lox/lox*^ mice is attributed to alterations in osteoblastic *Vdr* expression. Using these osteoblast-specific *Vdr*^*−/−*^ and *Vdr*^*ΔAF2*^ mice, we show that the osteoblast-specific loss of VDR signaling or coactivator-independent VDR signaling results in increased bone mass. In agreement with previous publications,^9,30,31^ these data confirm the importance of coactivator-dependent VDR signaling in osteoblasts and shows that coactivator-independent VDR signaling is not important in these cells. Hence, the coactivator-independent effects responsible for the severe bone phenotype of *Vdr*^*ΔAF2*^ mice are likely its negative effects on mineral (re)absorption, which result in persistent hypocalcemia and hypophosphatemia even on the rescue diet, along with chronically elevated PTH levels. Of note, previous research showed that *Vdr*^*−/−*^ and *Vdr*^*ΔAF2*^ mice on the rescue diet have significantly lower circulating levels of 25(OH)D_3_ and higher levels of 1,25(OH)_2_D_3_.^20^ However, both strains are unresponsive to co-activator-dependent 1,25(OH)_2_D_3_ signaling. Interestingly, the rescue diet is able to rescue the bone phenotype of *Vdr*^*−/−*^ mice but not that of *Vdr*^*ΔAF2*^ mice. Remarkably, renal calcium and phosphate conservation was lower in *Vdr*^*−/−*^ mice compared to *Vdr*^*ΔAF2*^ mice on the rescue diet. A possible explanation for this phenotypic difference in renal handling is VDR-mediated repression on calcium and phosphate reabsorption, a function that is lost in *Vdr*^*−/−*^ mice. The persistent calcium and phosphate deficits in *Vdr*^*ΔAF2*^ mice likely impair calcium and phosphate deposition into the osteoid matrix produced by osteoblasts. The number of osteoblasts seems to have increased in *Vdr*^*ΔAF2*^ mice as observed on Osx-labeled bone sections. As we did not observe differences in osteoblast numbers in osteoblast-specific *Vdr*^*ΔAF2*^ mice, we assume that external regulators such as PTH are responsible for the observed increased osteoblast numbers in systemic *Vdr*^*ΔAF2*^ mice. Increased osteoblast numbers in turn result in excessive deposition of osteoid matrix and collagen fibers, which remain undermineralized as shown by Goldner, Masson trichrome and Von Kossa/calcein stainings and µCT analysis. In addition, chronically high levels of PTH have been associated with fibrous dysplasia in humans, a disorder where normal bone and bone marrow are replaced by fibrous tissue.^32,33^ Interestingly, immunohistochemical analysis revealed that this human phenotype resembles the fibroblast-loaded bone phenotype observed in *Vdr*^*−/−*^ mice on normal diet and *Vdr*^*ΔAF2*^ mice on both normal and rescue diets. In addition, high PTH levels can induce osteoclastogenesis, as previously reported in *Vdr*^*−/−*^ mice on a normal diet. However we did not observe significant changes in OC.S/BS.^34^

Since these bone defects are likely indirect consequences of the low mineral supply and high PTH levels, we hypothesized that the main effects of coactivator-independent VDR signaling reside in the intestines or kidneys, negatively affecting mineral (re)absorption. Therefore, we performed RNA-seq studies in intestine and kidney of mice on the rescue diet to expose genes that are responsible for the persistent hypocalcemia and hypophosphatemia in *Vdr*^*ΔAF2*^ mice. In our RNA-seq studies, duodenal tissue was used because of the active transcellular calcium absorption and high *Vdr* expression within this proximal part of the intestine.^8^

Interestingly, expression of the calcium transporters *Trpv6* and *S100g* was significantly lower in *Vdr*^*ΔAF2*^ than in *Vdr*^*−/−*^ mice, suggesting that the VDR does not only induce these transporters but can also repress them. Repression of *Trpv6* and *S100g* might contribute to the more severe hypocalcemia and rickets phenotype observed in *Vdr*^*ΔAF2*^ mice. However, previous studies using *Trpv6/S100g* double knockout mice have shown that both transporters are important but not crucial factors in maintaining normal serum calcium levels, suggesting other transporters are to be identified.^35,36^ Therefore, we searched for other genes that might contribute to the observed phenotype in *Vdr*^*ΔAF2*^ mice focusing on genes that resembled the expression pattern of *Trpv6* and *S100g*, which were significantly more downregulated in *Vdr*^*ΔAF2*^ than in *Vdr*^*−/−*^ mice. We identified genes involved in mineral ion transport (*Car7*, *Cabp1*, and *Wdr72* in duodenum; *Slc22a27* and *Slc13a1* in kidney) and will investigate the physiological relevance of these genes in the future.

In conclusion, deletion of the VDR AF2 domain completely impairs ligand-dependent coactivator binding, but retains AF2-independent co-repressor binding. Here, we demonstrate the negative impact of these repressive actions in duodenum and kidney and its implications on calcium, phosphate, and bone homeostasis, evidenced by the more extremely impaired mineral homeostasis and bone phenotype observed in *Vdr*^*ΔAF2*^ mice compared to that in *Vdr*^*−/−*^ mice. We demonstrate that this bone phenotype is not mediated by coactivator-independent actions directly in bone cells, further emphasizing its role in intestine and kidney.

In addition, the *Vdr*^*ΔAF2*^ phenotype is also largely unresponsive to a diet high in calcium, phosphate, and lactose. Finally, based on this mouse model, we propose a list of potentially important, new, repressed VDR target genes in the interrelation between inorganic ions and calcium/phosphate (re)absorption. Trans-repression assays are currently being developed in our lab to further assess repressive VDR signaling in vitro.

## Materials and methods

### Nuclear receptor activity profiling (NAPing)

African green monkey kidney fibroblast-like COS-1 cells (8 × 10^6^ cells) were seeded in T175 flasks in DMEM supplemented with 10% fetal bovine serum (FBS), 2 mmol/L GlutaMax, 100 U/mL penicillin and 100 µg/mL streptomycin (all from Thermo Fisher Scientific). After 24 h, cells were transfected with 85 µg his-tagged *Vdr*^+/+^ or *Vdr*^*∆AF2*^ expression constructs. The next day, cells were scraped and pelleted. Cell lysates were used for the NAPing assay at Precision Medicine Lab (the Netherlands), as described previously.^37^ Briefly, each sample was incubated with vehicle (DMSO) or 1,25(OH)_2_D_3_ (Merck, 10^−7 ^mol/L) and processed on a chip platform containing 101 immobilized peptides with coregulator-derived LxxLL motifs. Subsequently, the binding of VDR^+/+^ and VDR^∆AF2^ proteins to each individual coregulator peptide was quantified using anti-his-tag antibodies. After normalization for protein input, the signal-minus background value of four technical replicates was used as the quantitative parameter of receptor-coregulator binding.

### Luciferase transactivation assay

Exponentially growing COS-1 cells were seeded at 13 000 cells per well in 24-well plates in DMEM supplemented with 10% FBS, 100 U/mL penicillin, 100 µg/mL streptomycin and 2 mmol/L GlutaMax. After 24 h, cells were transfected, using X-tremeGENE HP transfection reagent (Merck), with expression vectors for VDR^+/+^, VDR^*−/−*^ or VDR^ΔAF2^ and for RXR (50 ng each). Cells were co-transfected with a pGL3-Basic VDR luciferase reporter construct (250 ng; Promega), which contains four copies of a classical DR3-type VDRE and with pCDNA3.1(-)/Myc-His/*LacZ* (25 ng; Thermo Fisher Scientific). The next day, cells were stimulated with increasing doses of 1,25(OH)_2_D_3_ (10^−10 ^mol/L - 10^−7 ^mol/L) or vehicle (ethanol). After 24 h, luciferase activity was measured with a Firefly luciferase kit (Biotium, VWR, Avantor) and normalized to β-galactosidase activity, measured with the Galacto-Light Plus System (Applied Biosystems). Three technical replicates were performed for each condition.

### Transgenic mouse models

*Vdr*^*ΔAF2*^ mice were a kind gift of Dr. S. Kato (Institute of Molecular and Cellular Biosciences, University of Tokyo, Soma Central Hospital, Japan),^20^ and these mutant mice were obtained by introducing two stop codons at the beginning of exon 10, thereby transcriptionally deleting the 12 most C-terminal amino acids, comprising the AF2 domain of the VDR. *Vdr*^*−/−*^ mice were purchased from the Jackson laboratory (JAX stock #006133).^10^ These mice were generated by replacing exon 3, encoding the second zinc finger of the VDR, with a neomycin resistance gene. Genomic DNA of the three different mouse strains was sent for whole genome sequencing (Novogene, United Kingdom) to confirm the pre-determined mutations and to exclude off-target mutations.

Phenotypic analysis was performed on 8-week-old *Vdr*^*−/−*^, *Vdr*^*ΔAF2*^, and their *Vdr*^+/+^ littermates. Only female mice were included in this study as preliminary data on calcium and bone homeostasis showed no differences between male and female mice (data not shown). The genetic background of the mouse strains was characterized by a 384-SNP panel [Mouse Max Bax 384 SNP Panel (GM-SN-15), Charles River Genetic Testing Services Wilmington] on genomic DNA from tail cuts. Both strains were C57BL/6 J congenic ( ≥ 99.9% C57BL/6 J allelic profile percent match). Therefore, *Vdr*^+/+^ mice of both strains were pooled into one group. Genotyping was performed by polymerase chain reaction (PCR) with Go Taq G2 Flexi DNA polymerase (Promega) on genomic DNA from toe cuts. Heterozygous breeding pairs were phenotypically normal (data not shown) and maintained on a mouse breeding diet (V1124, Ssniff, Soest, Germany), whereas experimental mice were either weaned on a normal diet [1% calcium, 0.7% phosphate, 0% lactose (V1535, Ssniff)] or a high calcium diet (2% calcium, 1.25% phosphate, 20% lactose, Teklad custom diet TD.96348, Inotiv, West Lafayette, US), the latter referred to as “rescue diet”.

To generate osteoblast (mesenchymal cells) and osteoclast (myeloid cell lineage) specific *Vdr*^*−/−*^ and *Vdr*^*ΔAF2*^ mice, *Vdr*^*lox/*lox^ and *Vdr*^*lox/ΔAF2*^ mice were crossed with *Prrx1*-Cre mice or LysM-Cre mice, respectively. B6.Cg-Tg(*Prrx1-Cre*)1Cjt/J were obtained from the Jackson Laboratory and *LysM-Cre* breeding couples were provided by Prof. R. Brandes, Vascular Research Center, Frankfurt, Germany.^38^ The *Prrx1* promoter is expressed in fibroblasts, adipocytes, osteocytes and osteoblasts, whereas the *LysM* promoter is expressed in monocytes, granulocytes, macrophages and osteoclasts. Phenotypic analysis was performed on 8-week-old female *Prrx1/LysM Cre+ Vdr*^*lox/lox*^ and *Vdr*^*lox/ΔAF2*^ mice and their *Prrx1/LysM Cre-* littermates. Experimental mice were weaned on a normal calcium diet [Ssniff (V1535-000)]. To generate *Vdr*^*-/ΔAF2*^ mice, heterozygous *Vdr*^*+/-*^ mice were crossed with heterozygous *Vdr*^*+/ΔAF2*^ mice.

To analyze calcium apposition in bone, calcein (16 mg/kg body weight; Merck) was administered via intraperitoneal injections 4 days and 1 day prior to sacrifice. All mice were housed in an animal facility with 12 h dark/light cycles and constant room temperature with food and water supplied *ad libitum*. All animal experiments were approved by the ethical committee of the KU Leuven (P188/2016).

### Western Blot analysis

Proteins were extracted from kidneys or from cell pellets with lysis buffer [50 mmol/L TrisCl (pH 8), 150 mmol/L NaCl, 0.1% sodium dodecyl sulfate (SDS), 1% NP40, 0.5% sodiumdeoxycholate]. Protein pellets were sonicated and protein concentrations were determined with the bicinchoninic acid assay (BCA) method (Pierce™ BCA Protein Assay Kit, Thermo Fisher Scientific) according to the manufacturer’s recommendations. Twenty μg (kidney samples) or thirty µg (osteoclast samples) was separated on 4%–12% Bis-Tris SDS-PAGE gels (Thermo Fisher Scientific) and transferred to nitrocellulose membranes (Amersham™ Protran®, GE Healthcare). Membranes were incubated overnight at 4 °C with a primary antibody to VDR (1:500, D2K6W, Cell Signaling Technology) or β-actin (1:5 000, Sigma Aldrich) and subsequently incubated for two hours with a horseradish peroxidase (HRP) conjugated secondary antibody (1:5 000, Cell Signaling Technologies). Protein bands were visualized using enhanced chemiluminescence as described by the supplier (Odyssey XF, Li-Cor).

Protein lysates of osteoblast cultures (2.5 µg) were loaded and analysed on the ProteinSimple Wes system (ProteinSimple, San Jose, CA, USA), according to the manufacturer’s instructions using primary antibodies against VDR (D2K6W, Cell Signaling Technology) or β-Tubulin (BSA Free, NB600, Novus Biologicals).

### Serum and urine biochemistry

At 8 weeks of age mice were transferred to individual metabolic cages (Tecniplast, Buguggiate, Italy) to obtain 24 h urine collections and monitor food and water intake. Serum was collected at sacrifice. Urine and serum calcium (OSR60117), phosphate (OSR6122), and creatinine (OSR6178) concentrations were measured with a Beckman Colter DxC700AU chemistry analyzer (Analis, Suarlée, Belgium) and fractional urinary excretion of calcium and phosphate was calculated based on serum and urine calcium, phosphate, and creatinine levels. Serum concentrations of PTH (Quidel, San Diego, USA), FGF23 (Kainos,Tokyo, Japan), and CTx (Ratlaps, IDS, Frankfurt, Germany) were measured with an enzyme-linked immunosorbent assay (ELISA) according to the manufacturer’s instructions. Serum osteocalcin levels were measured using an in-house radio-immunoassay.^39^

### Micro-computed tomography (µCT)

The high resolution SkyScan 1272 system (Bruker, Belgium) was used to obtain ex vivo µCT images of the right tibia (source settings; 60 kV, 83 μA, 0.5 mm aluminum filter – scan settings; 5 µm pixel size, 180° angular rotation, 0.4° angular increment). Cone-beam reconstruction software (NRecon, Bruker) was used to reconstruct the scans based on the Feldkamp algorithm. These reconstructed datasets were used for 3D morphometric analysis using CT Analyzer software (CTAn, Bruker). Volumes of interest were selected at 2.25 mm – 2.75 mm (cortical) and 0.75 mm – 2 mm (trabecular) from a manually selected reference point beneath the growth plate, where the trabecular compartments converge into one compartment on a cross-sectional image. In systemic *Vdr*^*−/−*^ and *Vdr*^*ΔAF2*^ mice, distances from the reference point were adapted to the average tibia length of the strain to compensate for the large differences in tibia length between the strains (Table S1). The “automated trabecular and cortical bone selection method” (Bruker Method Note 008) was adapted and used to automatically select cortical regions of interest within the volumes of interest. Binary images were generated using a global thresholding of 80–255 determined to optimally separate bone and soft tissue. Analysis of these binary images was performed according to the guidelines of the American Society for Bone and Mineral Research^40^ and 3D models were constructed with CTvox software (Bruker, Belgium). Extreme trabecularization of the metaphyseal region disabled correct selection and analysis of the trabecular bone compartment in *Vdr*^*−/−*^ and *Vdr*^*ΔAF2*^ mice on the normal diet and in *Vdr*^*ΔAF2*^ mice on the rescue diet.

### Femur calcium content

Femurs were dried overnight at 100 °C and subsequently ashed for 5 h at 500 °C. Femur dry and ash weights were quantified. Ashes were dissolved overnight in 1 mL 1 mol/L HCl and diluted 1:50 in distilled water to measure the calcium concentration on a Beckman Colter DxC700AU chemistry analyzer.

### Histological analysis and bone histomorphometry

The dissected left tibiae were fixed overnight in 2% freshly prepared paraformaldehyde and subsequently decalcified in 0.5 mol/L ethylenediamine tetra-acetic acid (EDTA) in phosphate buffered saline (PBS, pH 7.4) for two weeks at 4 °C. Decalcified bones were dehydrated in graded ethanol concentrations and embedded in paraffin. Paraffin sections (4 µm) were dewaxed in xylene and rehydrated before immunohistochemical staining as described previously.^41^ Briefly, hematoxylin (Prosan) and eosin (0.6% eosin Y, Merck; 1% phloxine B, 2% orange G, Merck; H&E) staining was used to analyze general morphology. TRAP staining was used to visualize osteoclasts in the trabecular bone and its quantification was based on averaged values of two regions of interest within the secondary spongiosa of three slides per tibia, sectioned with 160 µm intervals. Masson trichrome staining (Merck HT15-1KT) was used to visualize collagen apposition. Vimentin - Osx co-staining was used to visualize fibroblasts and osteoblast lineage cells, respectively. Antigens were retrieved by heat induction (95 °C, pH 6) in sodium citrate buffer (pH 6, 1 mol/L HCl) and blocked in TNB blocking buffer [0.1 mol/L Tris-HCl (pH 7.5), 0.15 mol/L NaCl, 0.05% Tween 20, 20% normal serum]. Blocked sections were incubated overnight with anti-Vimentin primary antibody (1/50, AF2105, R&D systems) in TNB and subsequently incubated with rabbit-anti-goat-Biotin (Dako) secondary antibody. The signal was amplified by streptavidin-hrp (TSA), and visualized by Cyanine3 (Akoya biosciences - TSA fluorescein system) in amplification diluent (1/50, TSA) according to the manufacturers’ instructions. Next, sections were washed, blocked in TNB and incubated with anti-Osx primary antibody (1/500, sc-22536R, Santa Cruz Biotechnology). The next day, sections were incubated with the same rabbit-anti-goat-Biotin secondary antibody, amplified by streptavidin-hrp (TSA), and visualized by FITC (TSA) in amplification diluent (1/50, TSA). As a negative control, after Vimentin staining and visualization, sections were washed, blocked and incubated with rabbit-anti-goat-Biotin secondary antibody and fluorescently labeled with FITC (TSA) without prior incubation with anti-Osx primary antibody. No fluorescent FITC signal was observed after visualization by streptavidin amplification (TSA).

The dissected right tibiae were fixed in Burckhardt solution and these non-decalcified bones were embedded in methylmethacrylate (MMA). MMA sections (4 µm) were used for Von Kossa staining to visualize mineral (calcium phosphate) apposition, for Goldner staining to visualize unmineralized bone (osteoid) and for calcein double labeling to visualize dynamic bone parameters (only quantifiable in bone specific mouse models). All stainings were imaged on the Axioscan 7 microscope slide scanner (Zeiss).

### Primary osteoblast and osteoclast cultures

Primary osteoblast cultures were isolated from calvarias of 3-day old *Prrx1-Cre*^±^*;Vdr*^*lox/lox*^ pups as described previously.^42^ Briefly, upon dissection, calvarias were digested in PBS containing 0,1% (w/v) collagenase type II and 0,2% (w/v) dispase type II (both from Gibco, Life Technologies) for six times 10 min at 37 °C. All digest fractions, except for the first fraction, were harvested, spinned down (10 min 1 000 r/min), resuspended in complete αMEM medium (with 10% FBS and 100 U/mL penicillin/streptomycin) and seeded in 6-well plates. When confluent after the first passage, cells were either lysed for protein isolation or treated with vehicle vs. 1,25(OH)_2_D_3_ (10^−7 ^mol/L) for 24 h and lysed for RNA-isolation.

Primary osteoclast cultures were isolated and grown as described previously.^38^ Briefly, bone marrow cells were isolated from 8-week-old *LysM Cre* ^±^*;Vdr*^*lox/lox*^ mice and cultured overnight in complete αMEM medium, containing 10 ng/mL macrophage-colony stimulating factor (M-CSF; R&D Systems, Abingdon, UK). The next day, bone marrow-derived hematopoetic cells (the non-adherent cells) were harvested and seeded at a cell density of 125 000 cells/mL in complete αMEM, containing 20 ng/mL M-CSF and 100 ng/mL receptor activator of nuclear receptor factor κB (RANKL, Peprotech, Rocky Hill, NJ, USA) and re-stimulated after 3 days of culture. After 4 days of culture, cells were either lysed for protein isolation or treated with vehicle vs. 1,25(OH)_2_D_3_ (10^−7 ^mol/L) for 24 h and lysed for RNA-isolation (InnuPREP RNA isolation kit, Westburg).

### Quantitative real-time reverse-transcriptase polymerase chain reaction (qRT-PCR)

Tissues were isolated at sacrifice, snap-frozen in liquid nitrogen and stored at −80 °C until further processing. Tissues (kidney, duodenum, white adipose tissue, brown adipose tissue and growth plates) were homogenized (Precellys 24, Bertin technologies, France) in TRIzol Reagent (Invitrogen) and RNA was extracted according to the manufacturer’s instructions. For primary osteoblast and osteoclast cultures, cells were lysed and RNA was isolated with the InnuPREP RNA isolation kit (Westburg, Leusden, The Netherlands). One µg of RNA was converted into cDNA using oligo(dT) primers and SuperScript II reverse transcriptase (Invitrogen). Gene expression was assessed in triplicate by real-time qRT-PCR using Taqman Fast Universal PCR master mix (Applied Biosystems) for primers with probe or SYBR PCR master mix (Thermofisher scientific) for primers without probe, according to the supplier’s protocol. Threshold cycle (Ct) values were measured on a QuantStudio 3 (Applied Biosystems) and relative gene expression was calculated with the 2^−^^ΔΔCt^ method. Expression levels of the gene of interest were normalized to the average expression levels of the house keeping genes *β-actin* and hypoxanthine-guanine phosphoribosyltransferase (*Hprt*). Primer and/or probe sequences used are listed in Table S2.

### RNA sequencing

Kidney cortex and duodenum were isolated from 8-week-old female *Vdr*^+/+^ (*n* = 6), *Vdr*^*−/−*^ (*n* = 4), and *Vdr*^*ΔAF2*^ (*n* = 4) mice maintained on the rescue diet. RNA isolation was performed as described above. RNA integrity (RIN) was assessed with a Bioanalyzer 2100 (Agilent Technologies) and libraries were prepared with the LexoGen QuantSeq Library preparation kit by the Genomics Core (KU Leuven, Belgium). Samples were subsequently sequenced (50 bp with 5’ single end) on the Illumina HiSeq 4000. Averages of 4.3 × 10^6^ reads/sample (kidney) and 3.8 × 10^6^ reads/sample (duodenum) were obtained and reads were mapped against the *mus musculus* reference genome (mm10). Genes were considered expressed when the counts per million was 1 or higher in at least 10% of the samples. Raw sequencing data were normalized using the standard trimmed mean of *M*-values (TMM) normalization method. Differential gene expression was performed with Limma v3.34.9 (R/Bioconductor software package in Biomex v1.0-5).^43^ To visualize the volcano plots, a threshold fold change of 1.4 (Log_2_ fold change of 0.485) and a threshold *P* value of 0.05 (Log_10_ *P* value of 1.3) was applied.

### Statistical analysis

For the NAPing assay, coregulatory binding was represented on a Log _10_-fold change (LFC) scale or as absolute binding and a Student’s *t*-test was applied to compare the 1,25(OH)_2_D_3_-stimulated VDR binding level to that of the unstimulated (DMSO) control for each coregulator peptide. Signal intensity of the luciferase transfection assay was expressed as mean ± standard error of the mean (SEM) and a two-way ANOVA followed by Dunnett multiple comparison test was used to analyze the data. For the comparison of *Prrx1/LysM Cre*^*+*^ and *Cre*^*-*^ mice to validate the *Vdr* knockdown efficiency, a Student’s t-test was applied. For the in vivo experiments, one-way ANOVA followed by Tukey’s multiple comparisons test was used to compare the groups for all parameters which passed normality and equal variance tests. For parameters with abnormal distribution or unequal variance, the non-parametric Kruskal-Wallis test was used. All in vivo data were expressed as mean ± upper/lower quartile and whiskers depicting minimum and maximum values. Differences were considered significant at *P* < 0.05 and all analyses were performed with Prism 8.2.1 (Graphpad Software, La Jolla, CA, USA).

### Supplementary information

Supplementary figure 1

Supplementary figure 2

Supplementary figure 3

Supplementary figure 4

Supplementary figure 5

Supplementary table 1

Supplementary table 2

Overview of bone histology and microCT

Top 25 genes_counts and statistics

Supplementary figure and table Legends

## Data Availability

The raw (BAM files) and processed (raw count files) RNA-seq data are available on the GEO database (GSE260988).
