# Supplementary figures and images for: Coactivator-independent vitamin D receptor signaling causes severe rickets in mice, that is not prevented by a diet high in calcium, phosphate, and lactose
Source: Bone Res. 2024 Aug 20;12:44. doi: 10.1038/s41413-024-00343-7 (PMC11335873; doi:10.1038/s41413-024-00343-7)

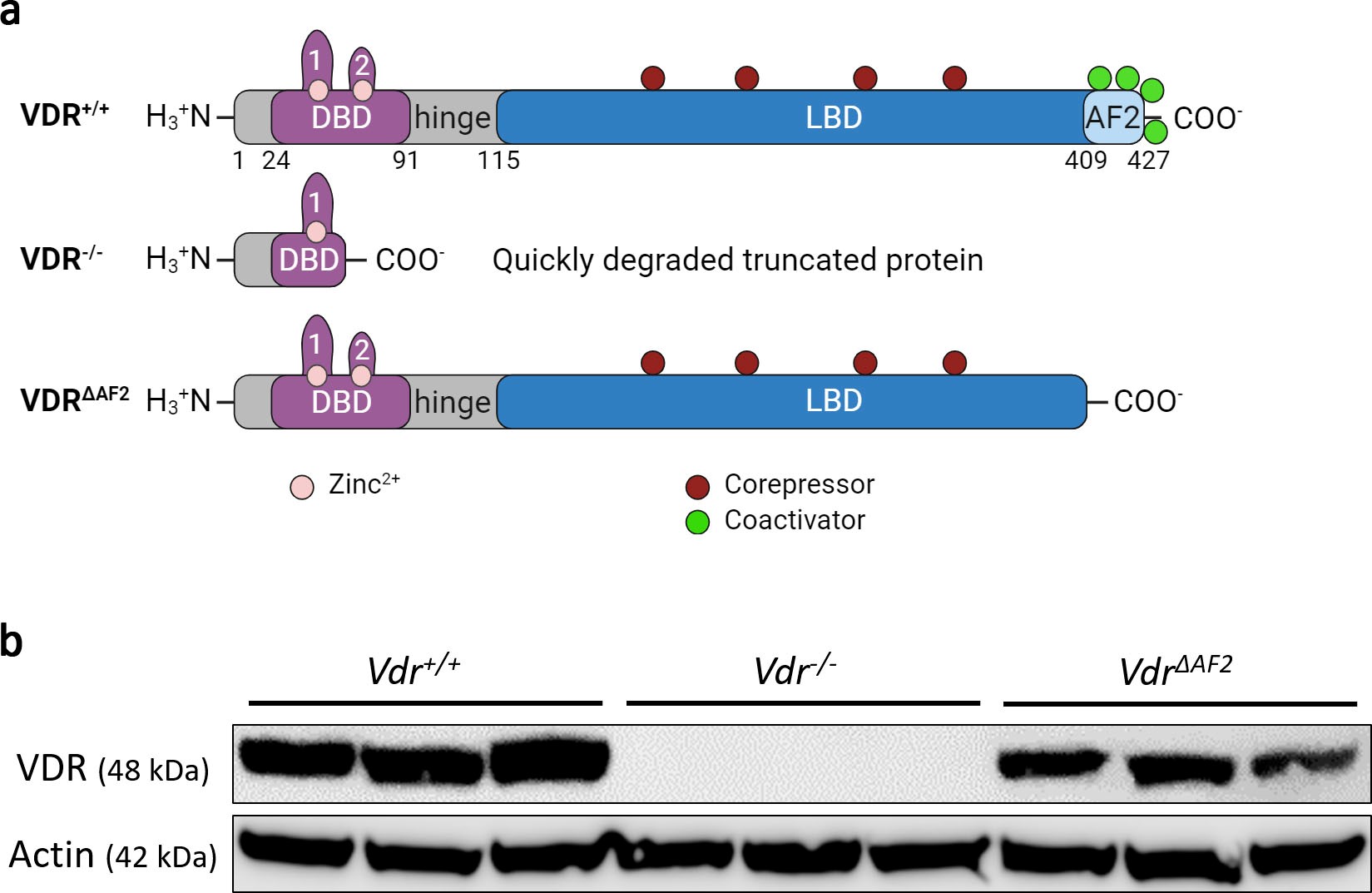

Supplement: Supplementary file 1 — Supplementary figure 1 [file 41413_2024_343_MOESM1_ESM.jpg]

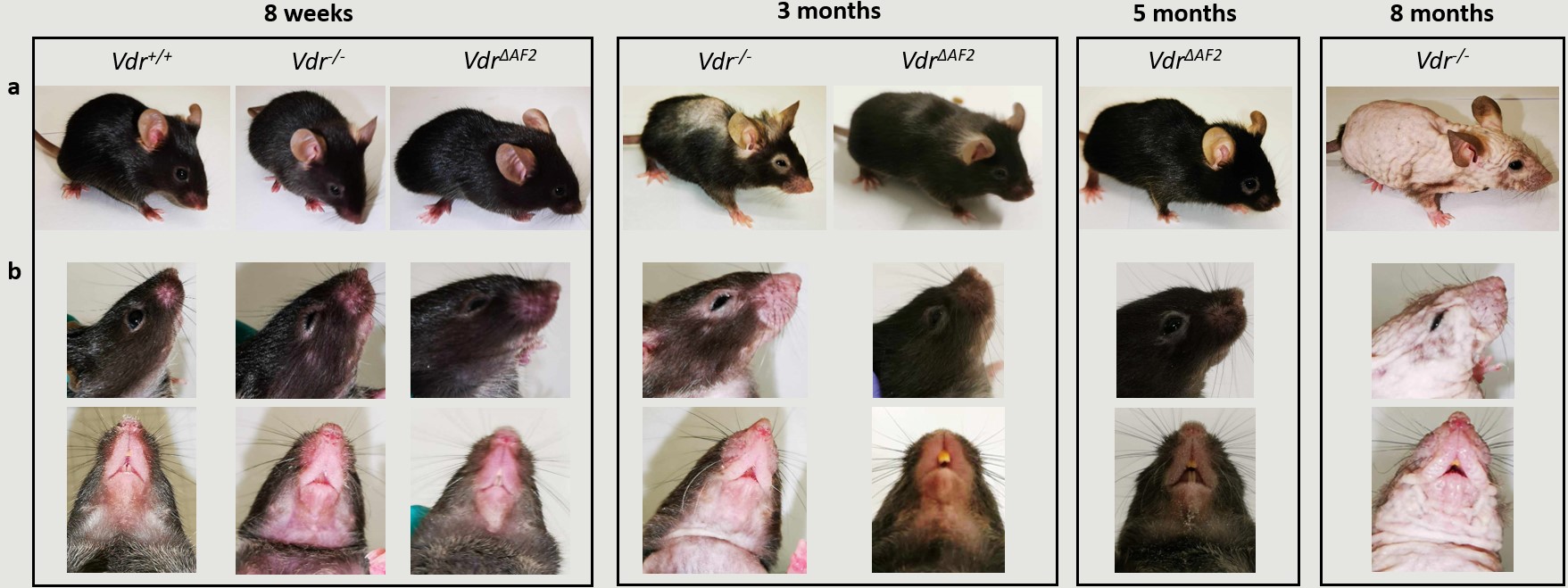

Supplement: Supplementary file 2 — Supplementary figure 2 [file 41413_2024_343_MOESM2_ESM.jpg]

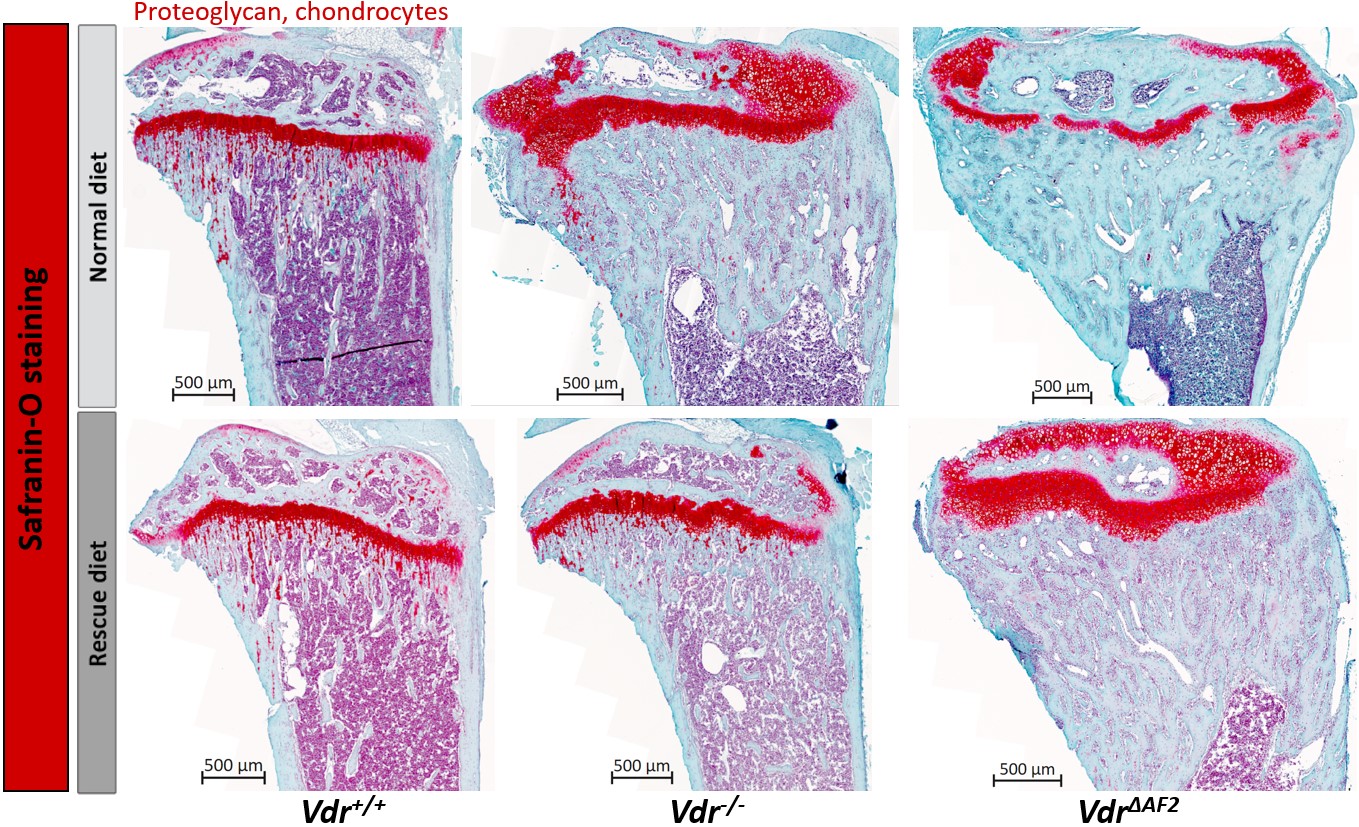

Supplement: Supplementary file 3 — Supplementary figure 3 [file 41413_2024_343_MOESM3_ESM.jpg]

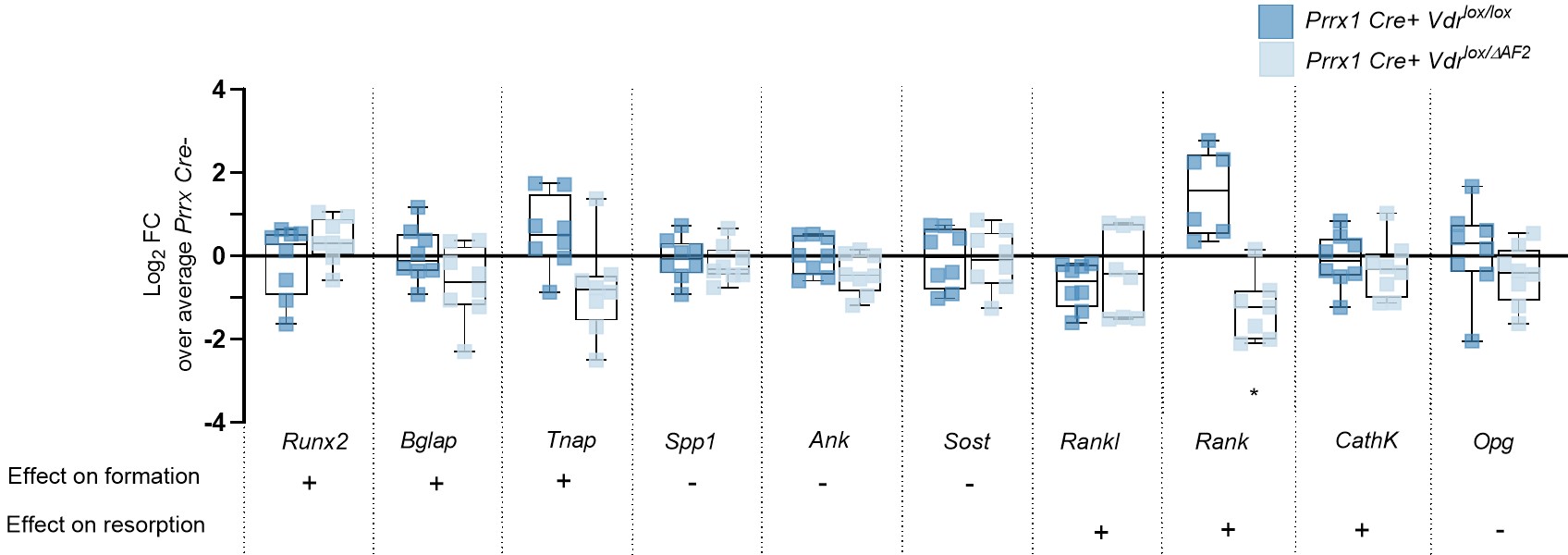

Supplement: Supplementary file 4 — Supplementary figure 4 [file 41413_2024_343_MOESM4_ESM.jpg]

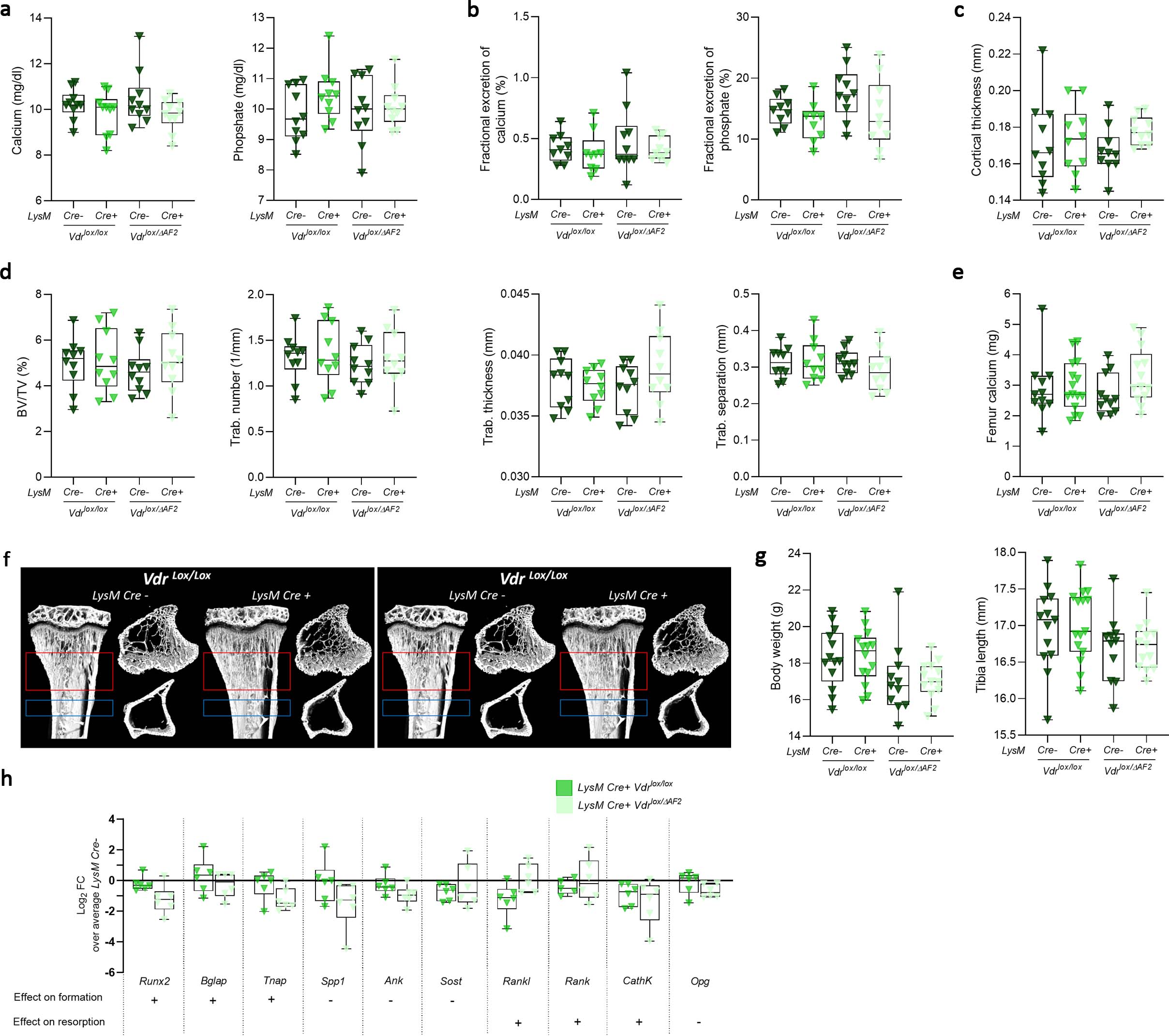

Supplement: Supplementary file 5 — Supplementary figure 5 [file 41413_2024_343_MOESM5_ESM.jpg]

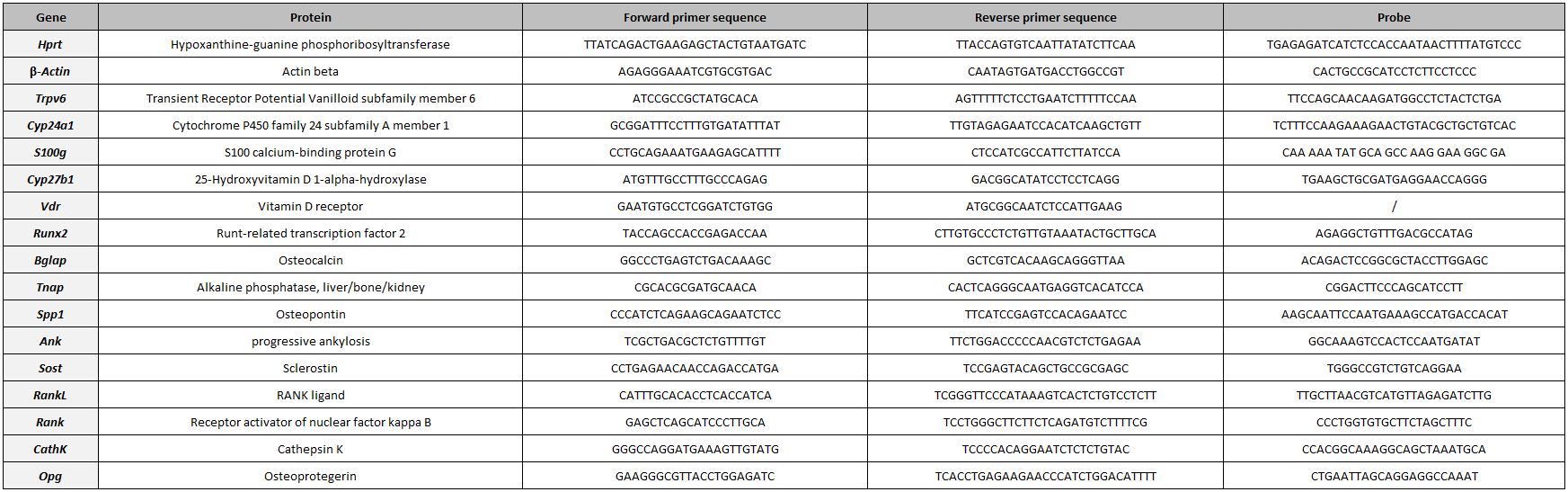

Supplement: Supplementary file 7 — Supplementary table 2 [file 41413_2024_343_MOESM7_ESM.jpg]

## Slide 1
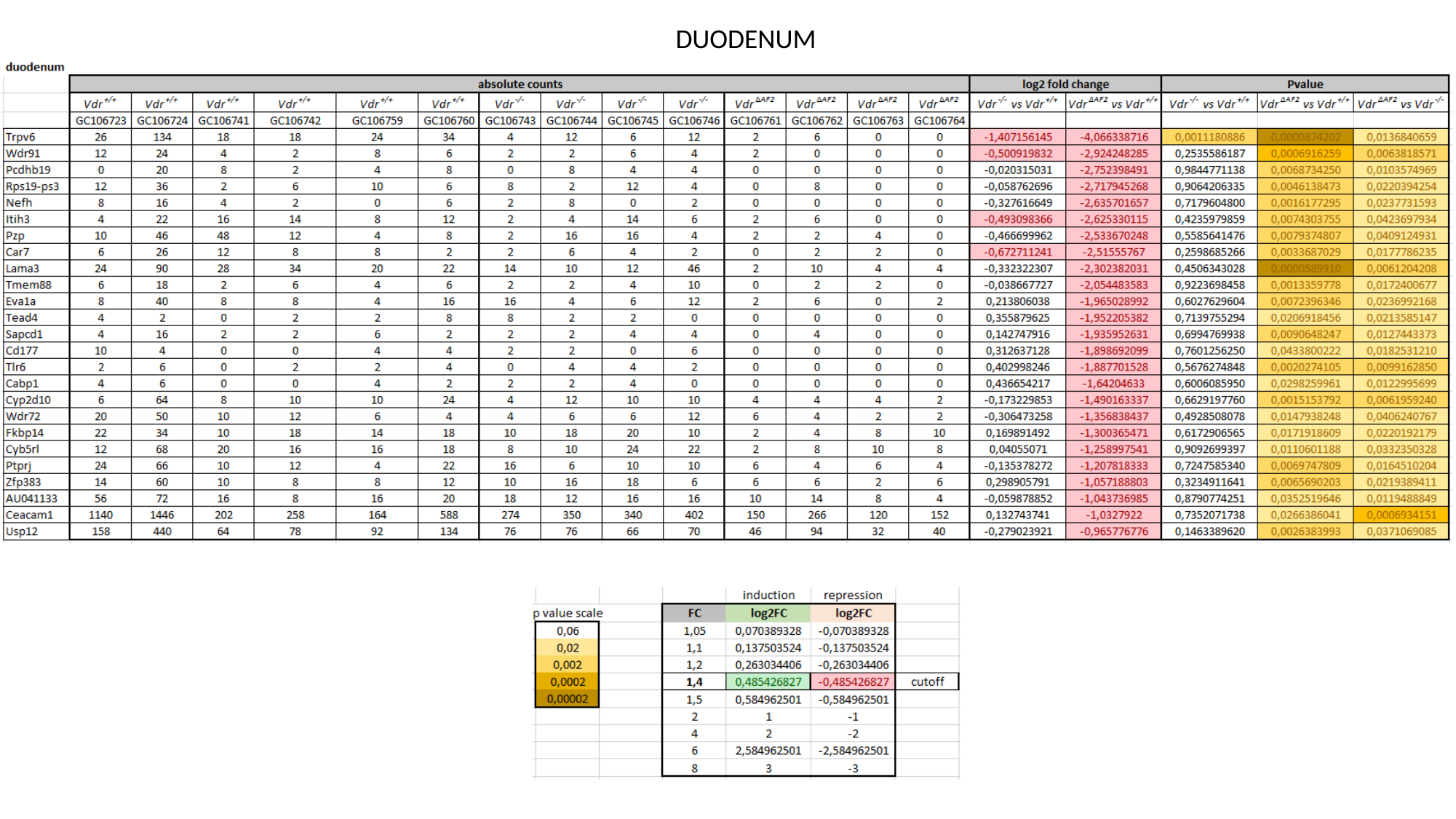

DUODENUM

## Slide 2
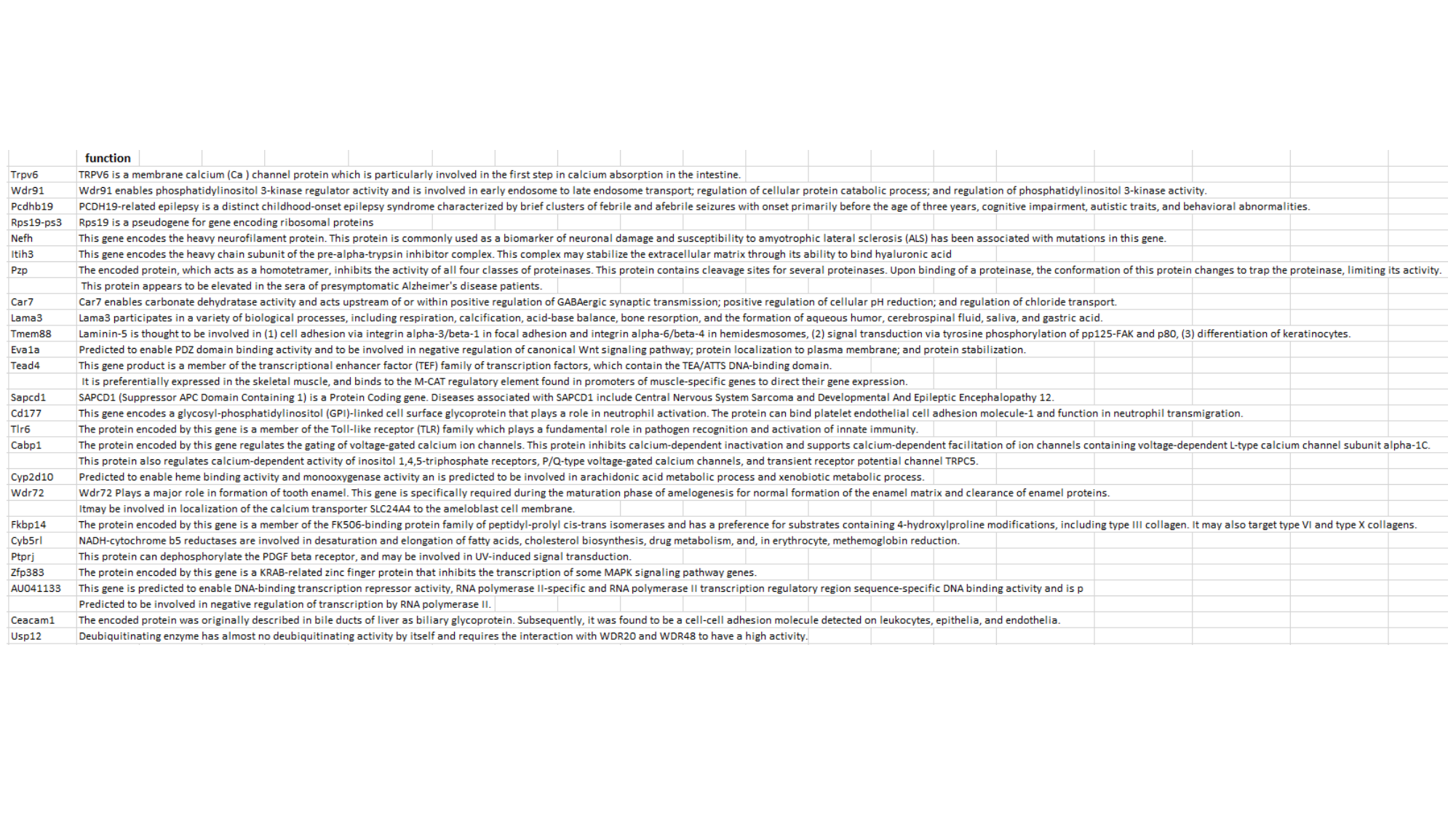

## Slide 3
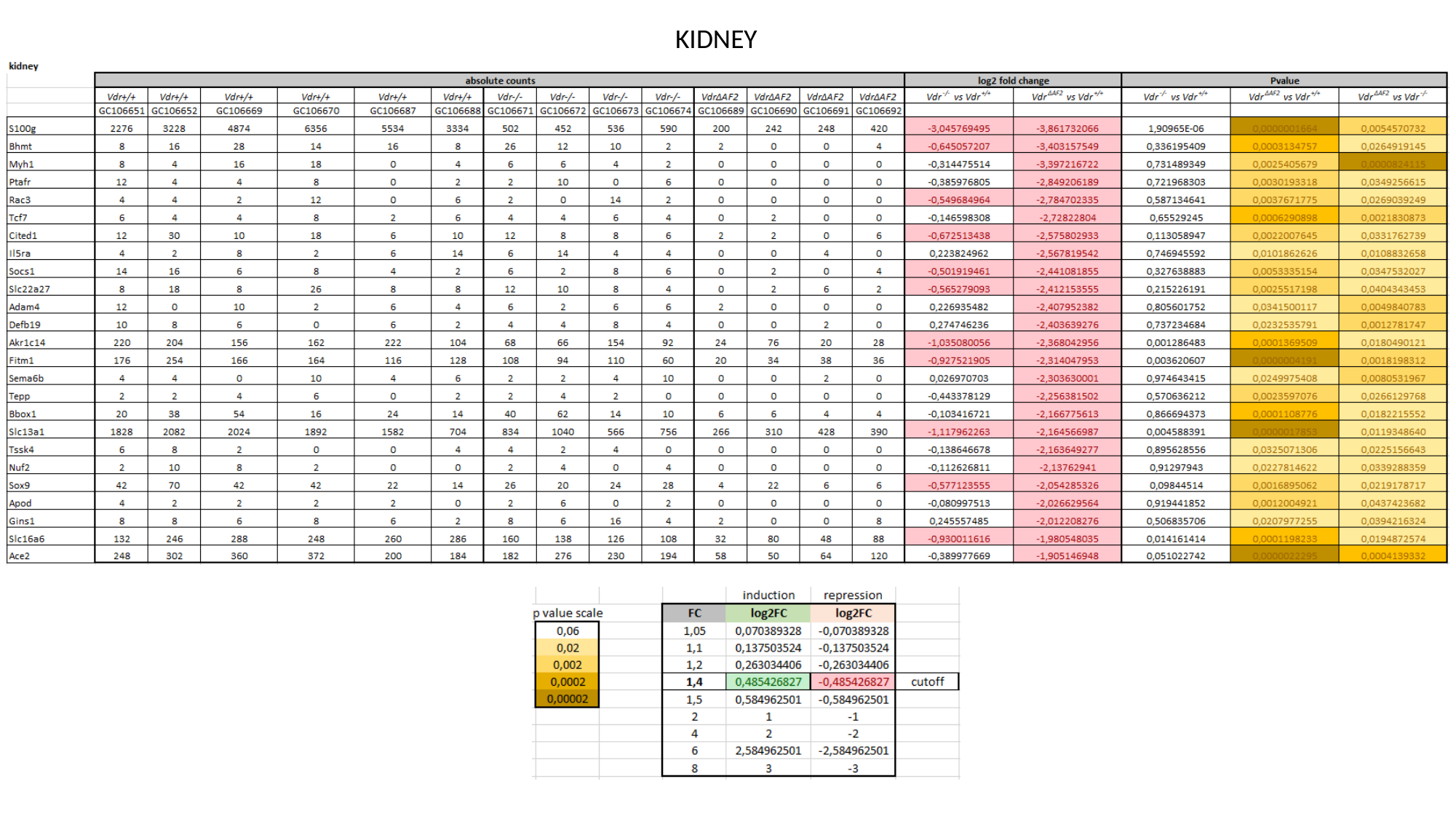

KIDNEY

## Slide 4
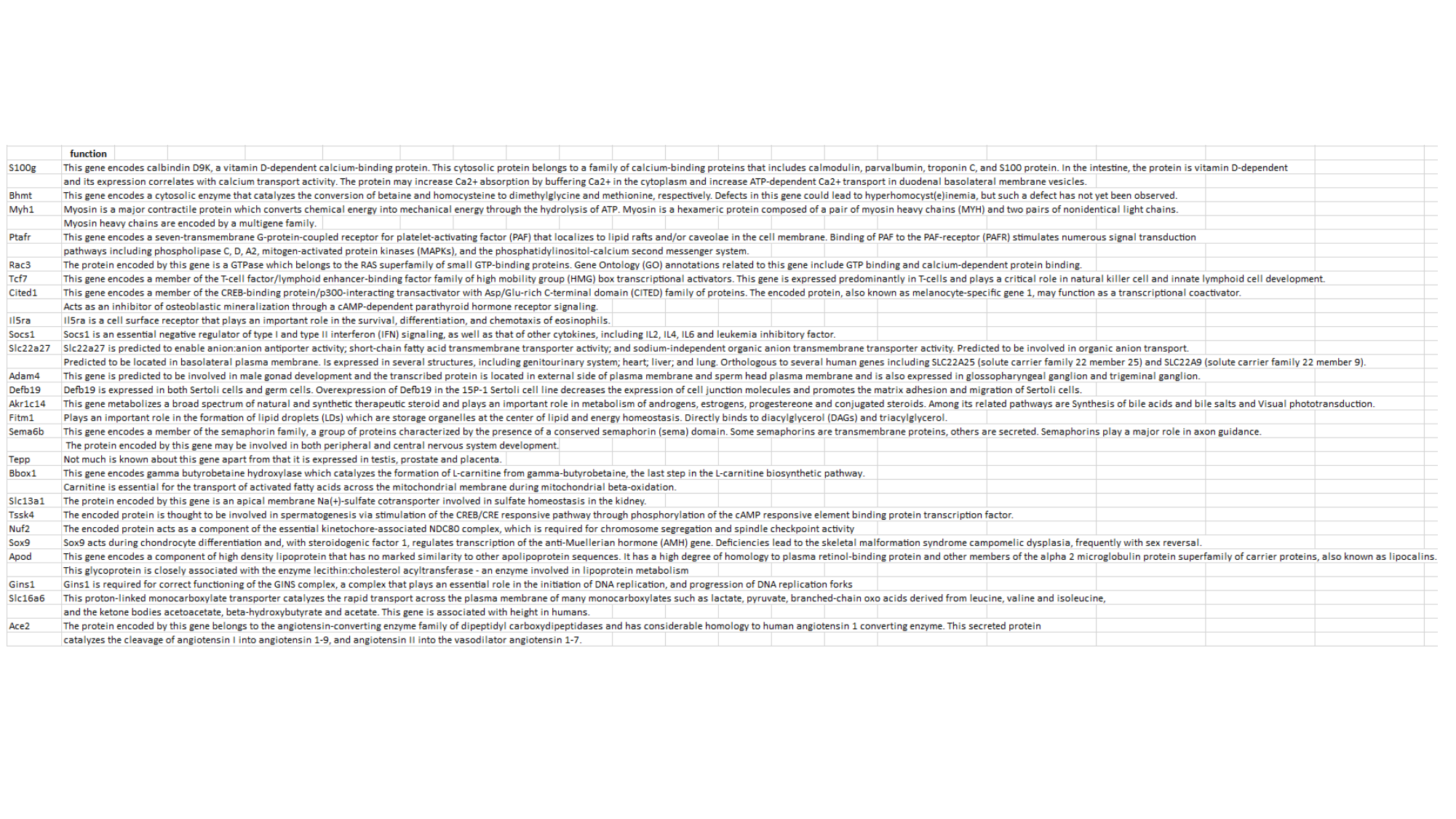

Supplement: Supplementary file 9 — Top 25 genes_counts and statistics [file 41413_2024_343_MOESM9_ESM.pptx]
