# Supplementary material for: Coactivator-independent vitamin D receptor signaling causes severe rickets in mice, that is not prevented by a diet high in calcium, phosphate, and lactose
Source: Bone Res. 2024 Aug 20;12:44. doi: 10.1038/s41413-024-00343-7 (PMC11335873; doi:10.1038/s41413-024-00343-7)

## Slide 1
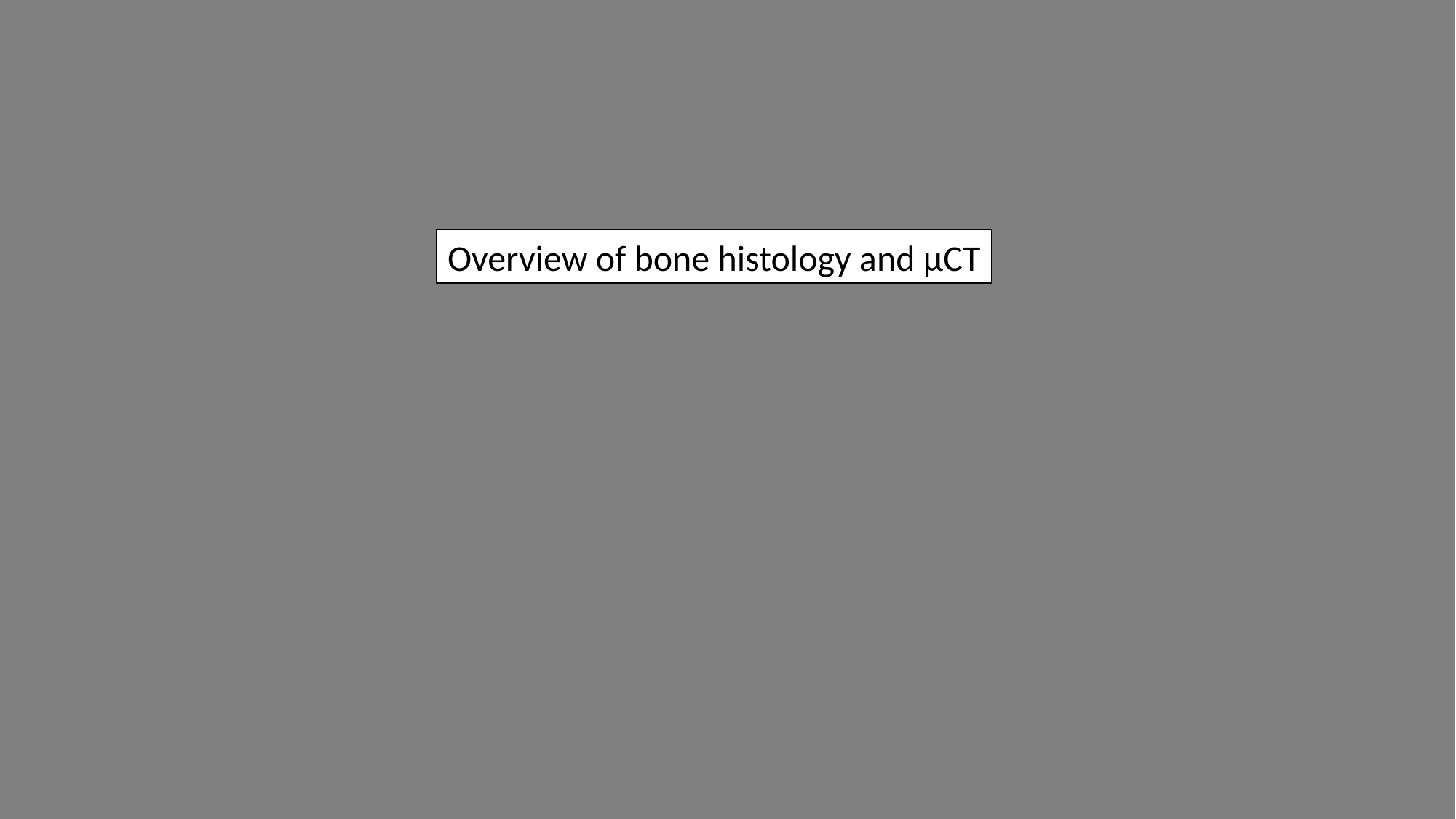

Overview of bone histology and µCT

## Slide 2
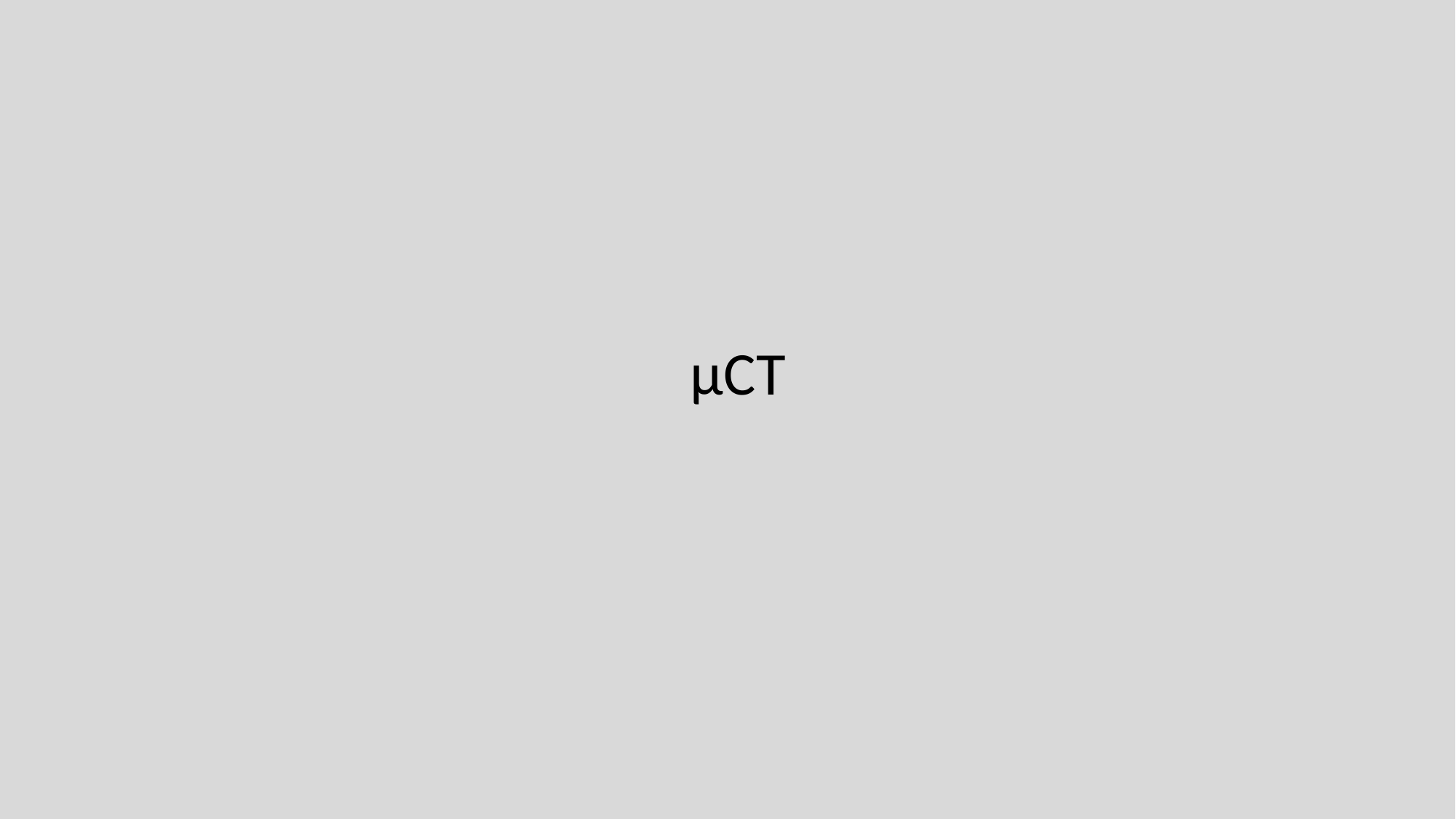

µCT

## Slide 3
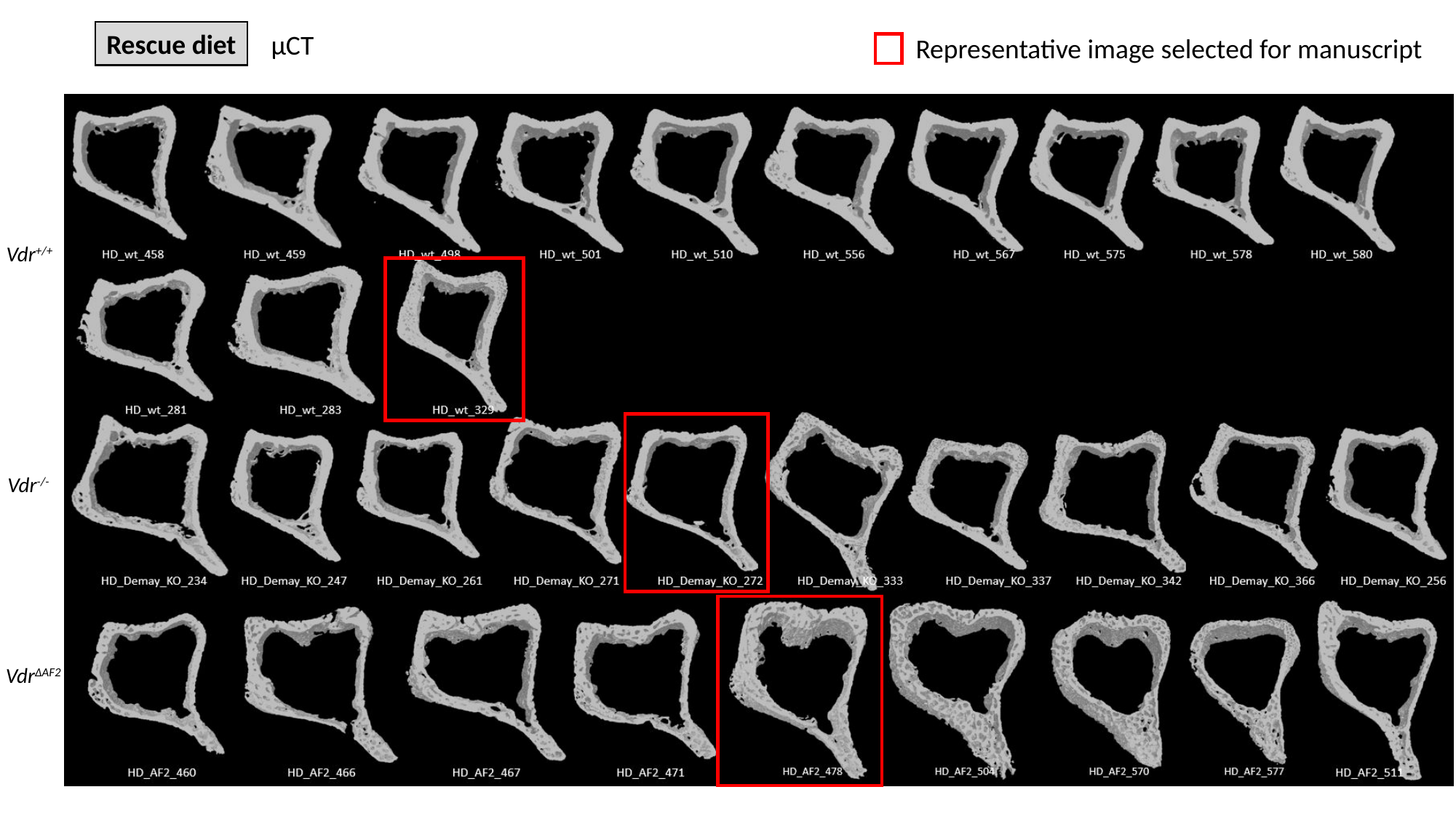

Rescue diet
µCT
Representative image selected for manuscript
Vdr+/+
Vdr-/-
VdrΔAF2

## Slide 4
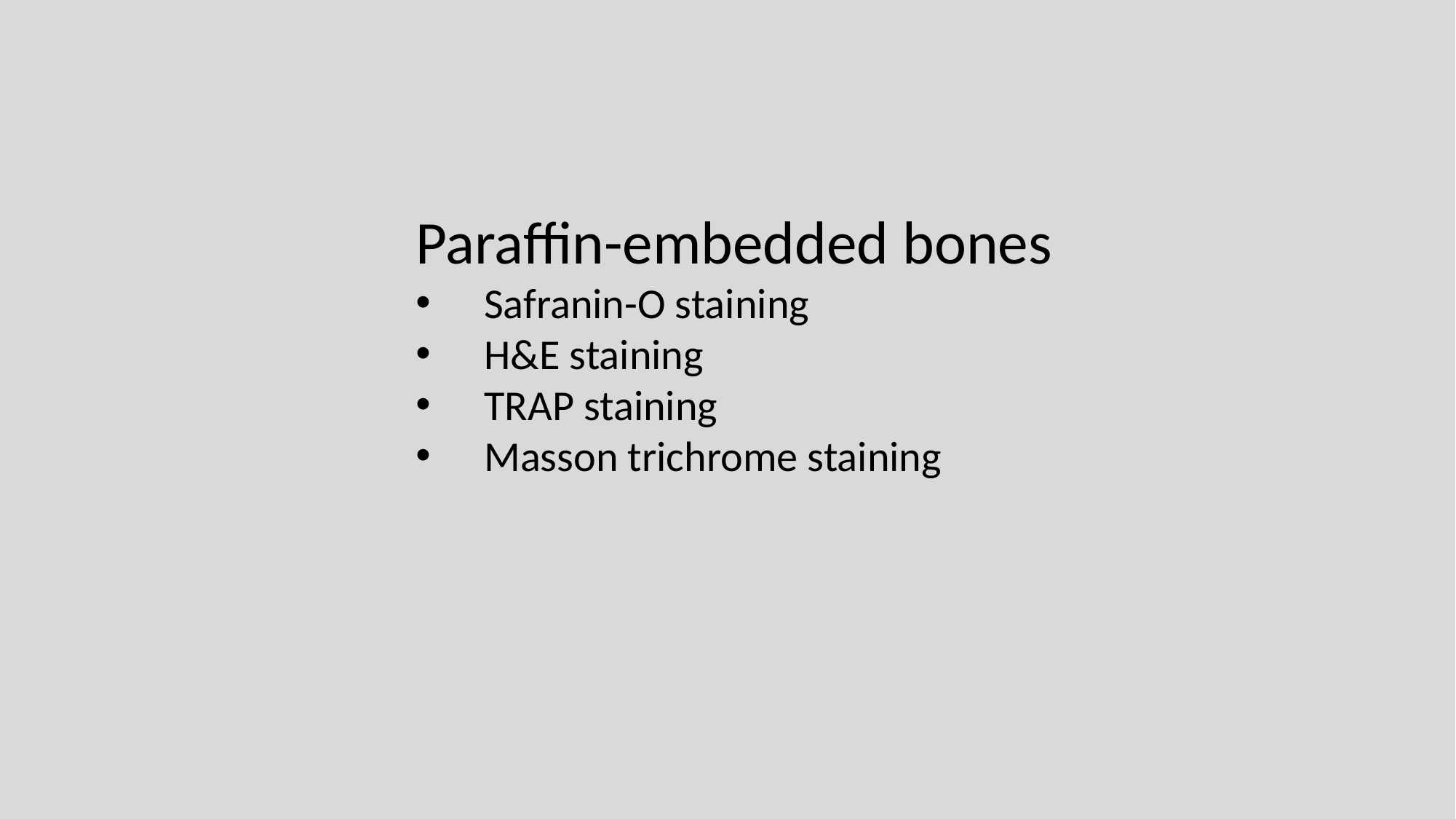

Paraffin-embedded bones
Safranin-O staining
H&E staining
TRAP staining
Masson trichrome staining

## Slide 5
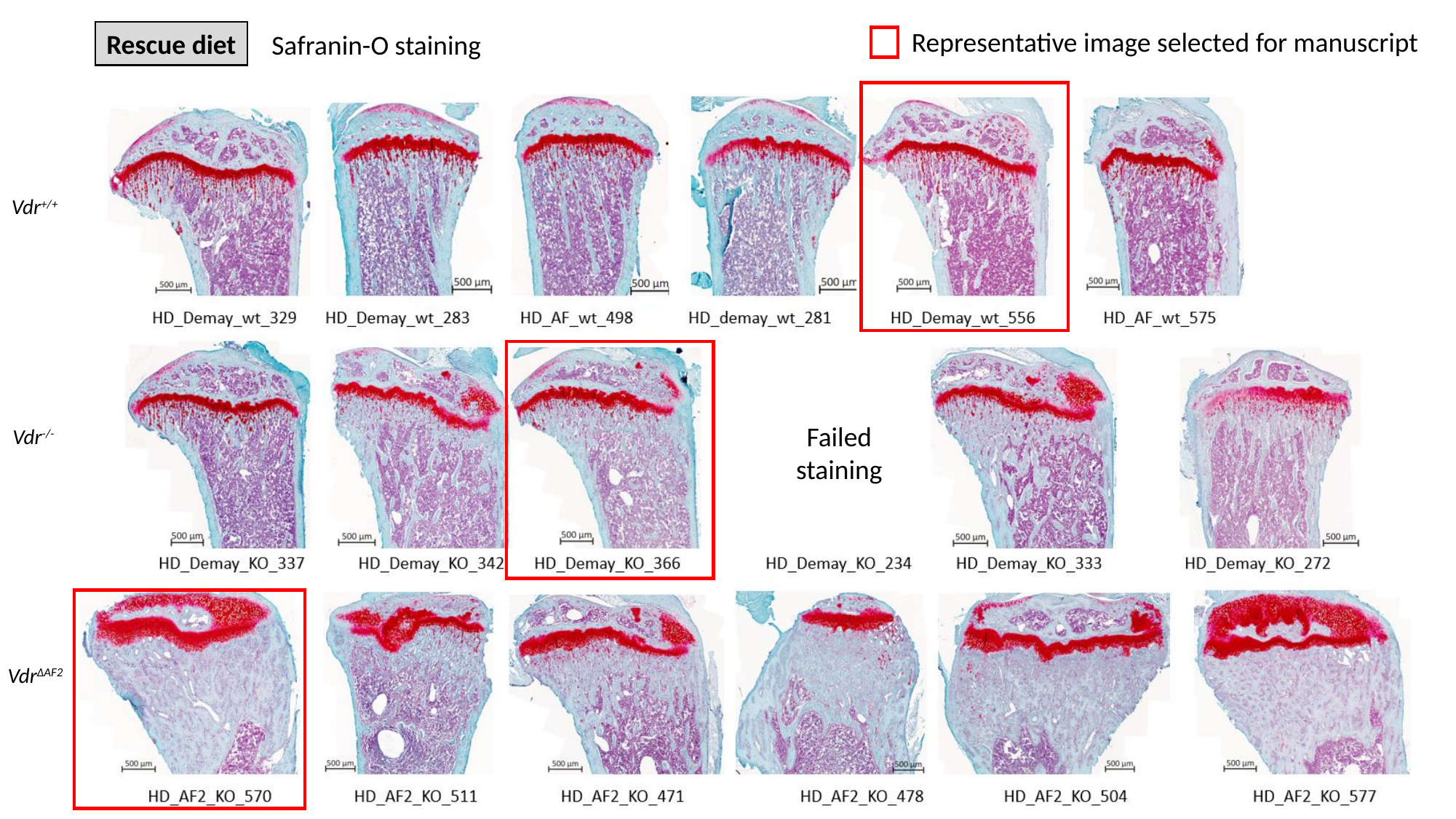

Representative image selected for manuscript
Rescue diet
Safranin-O staining
Failed staining
Vdr+/+
Vdr-/-
VdrΔAF2

## Slide 6
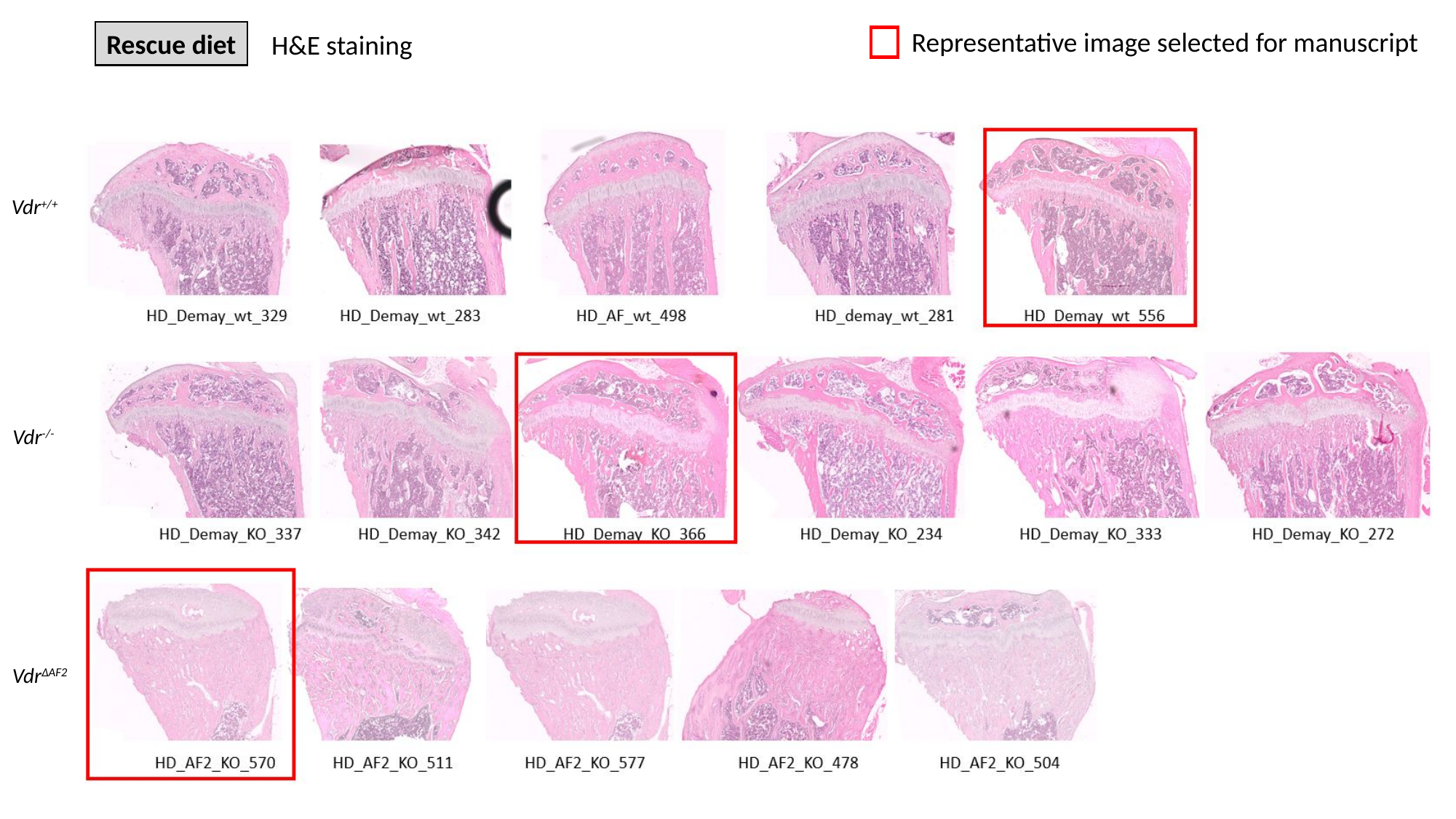

Representative image selected for manuscript
Rescue diet
H&E staining
Vdr+/+
Vdr-/-
VdrΔAF2

## Slide 7
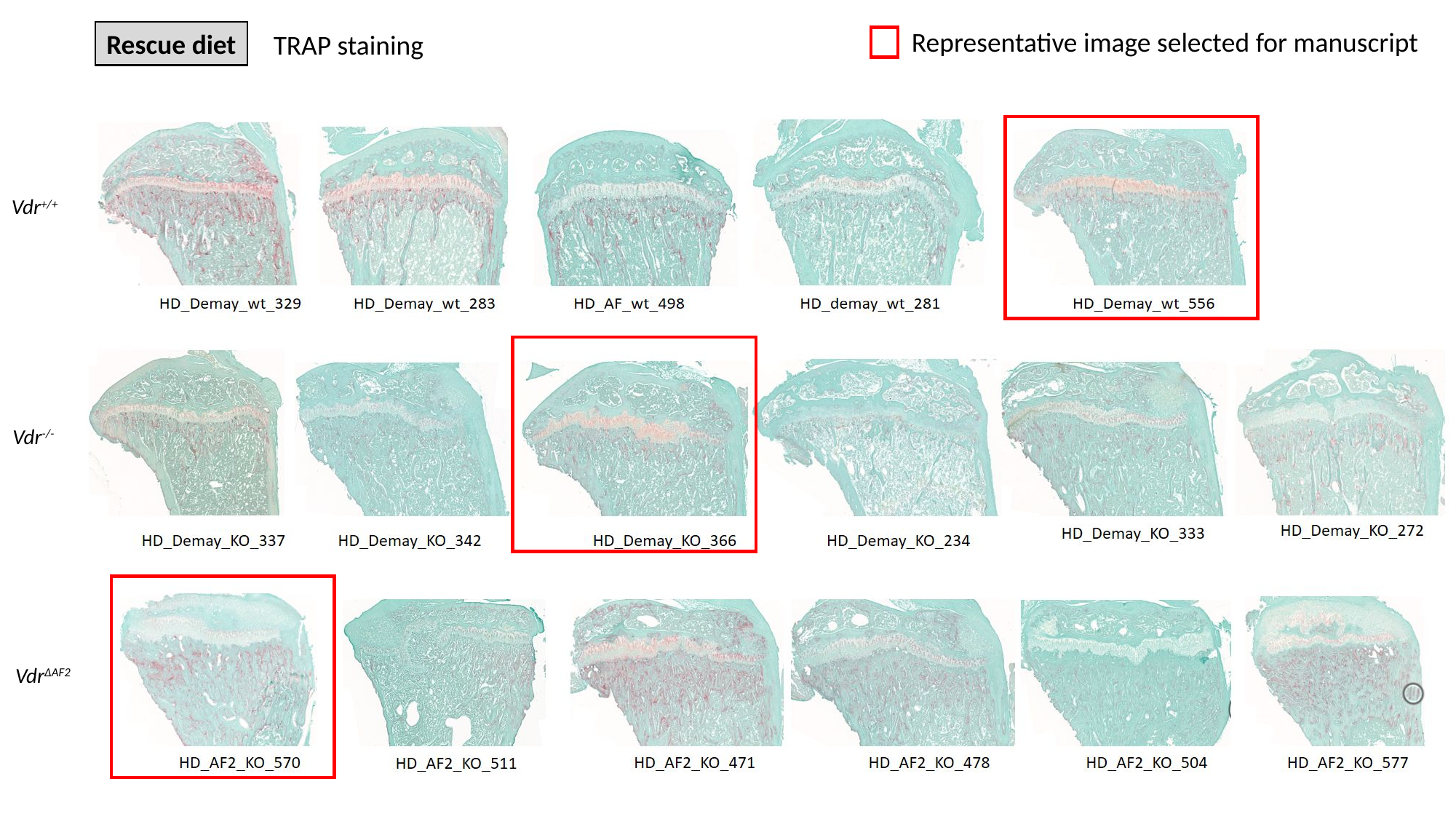

Representative image selected for manuscript
Rescue diet
TRAP staining
Vdr+/+
Vdr-/-
VdrΔAF2

## Slide 8
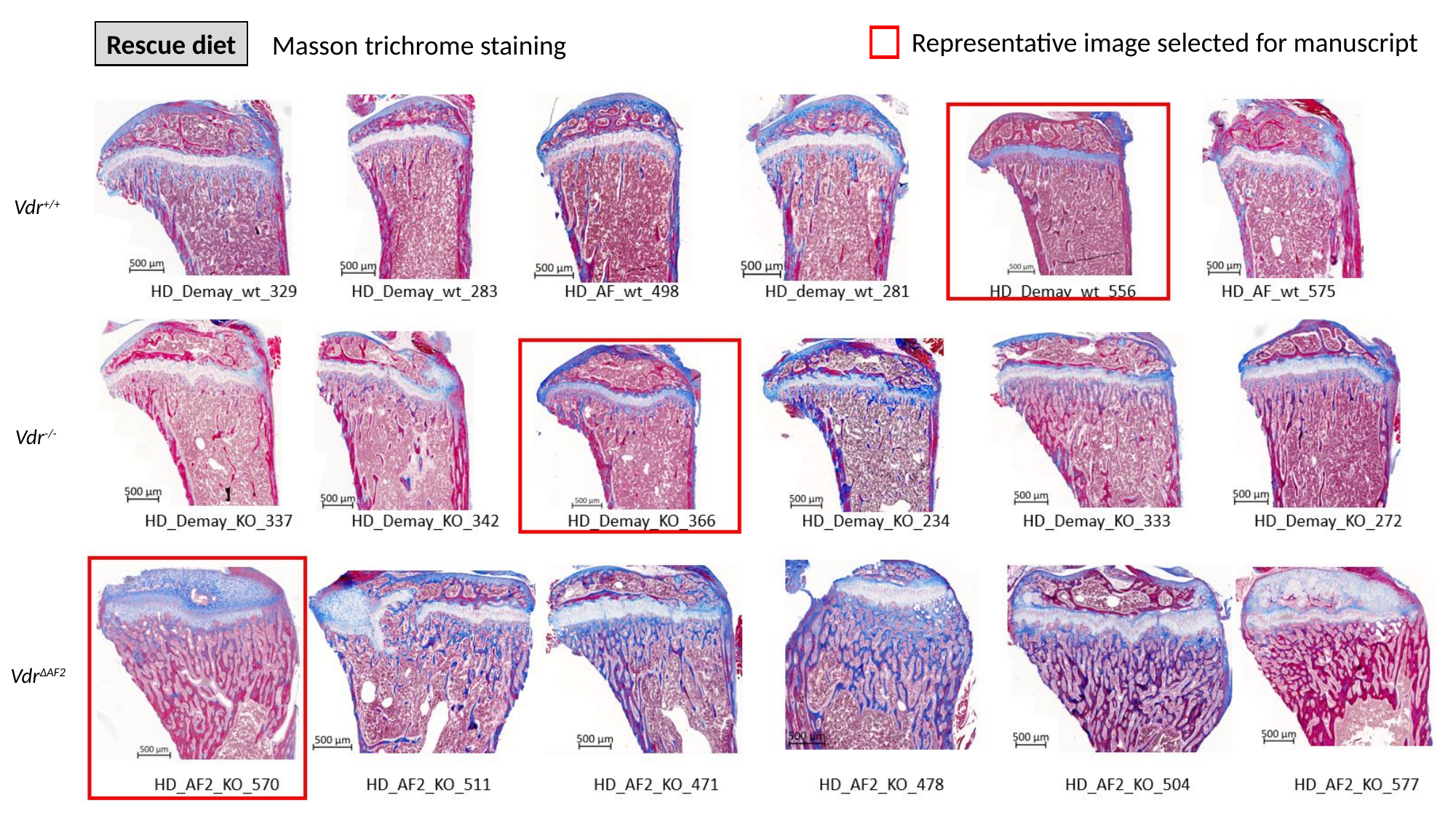

Representative image selected for manuscript
Rescue diet
Masson trichrome staining
Vdr+/+
Vdr-/-
VdrΔAF2

## Slide 9
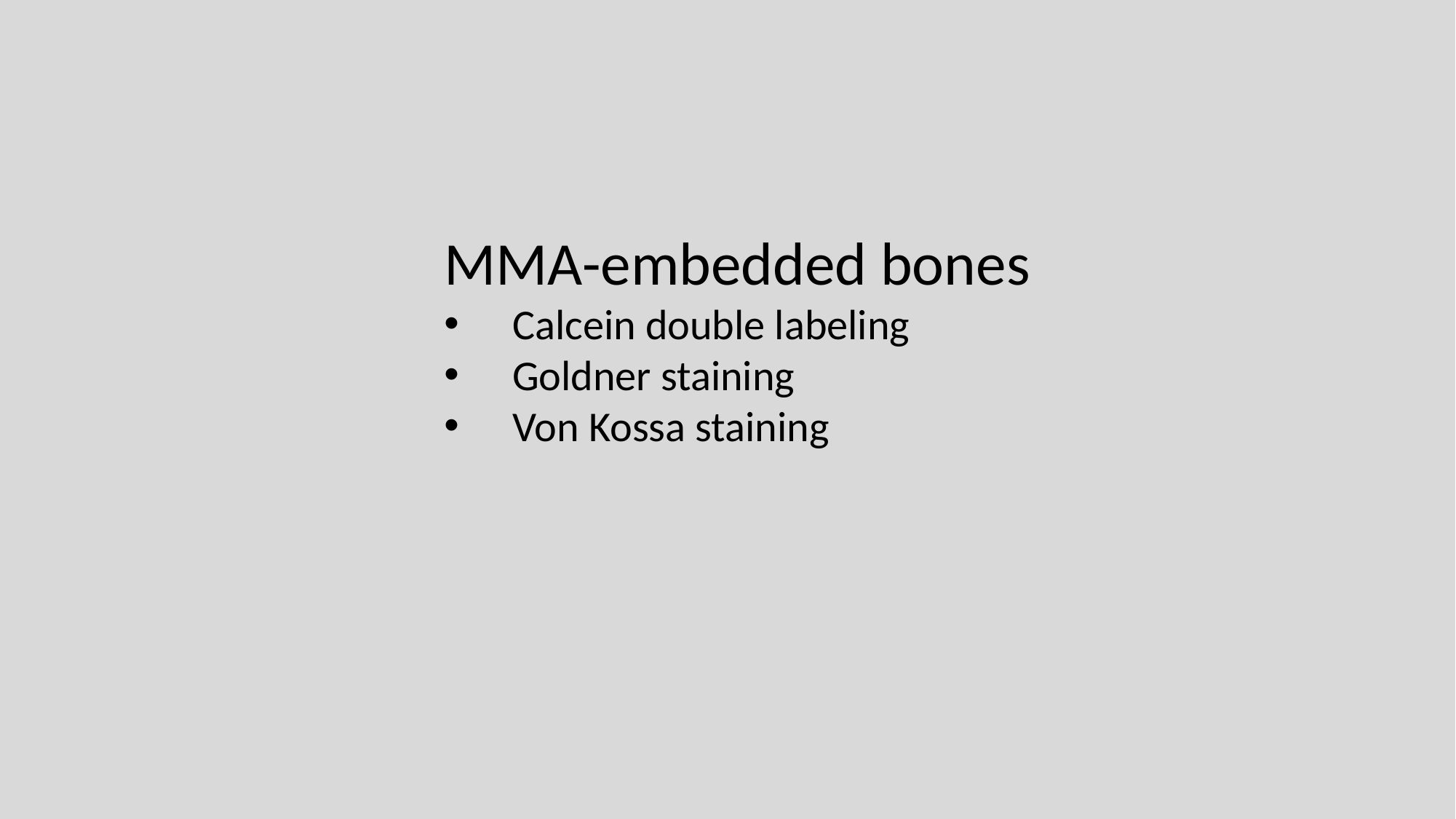

MMA-embedded bones
Calcein double labeling
Goldner staining
Von Kossa staining

## Slide 10
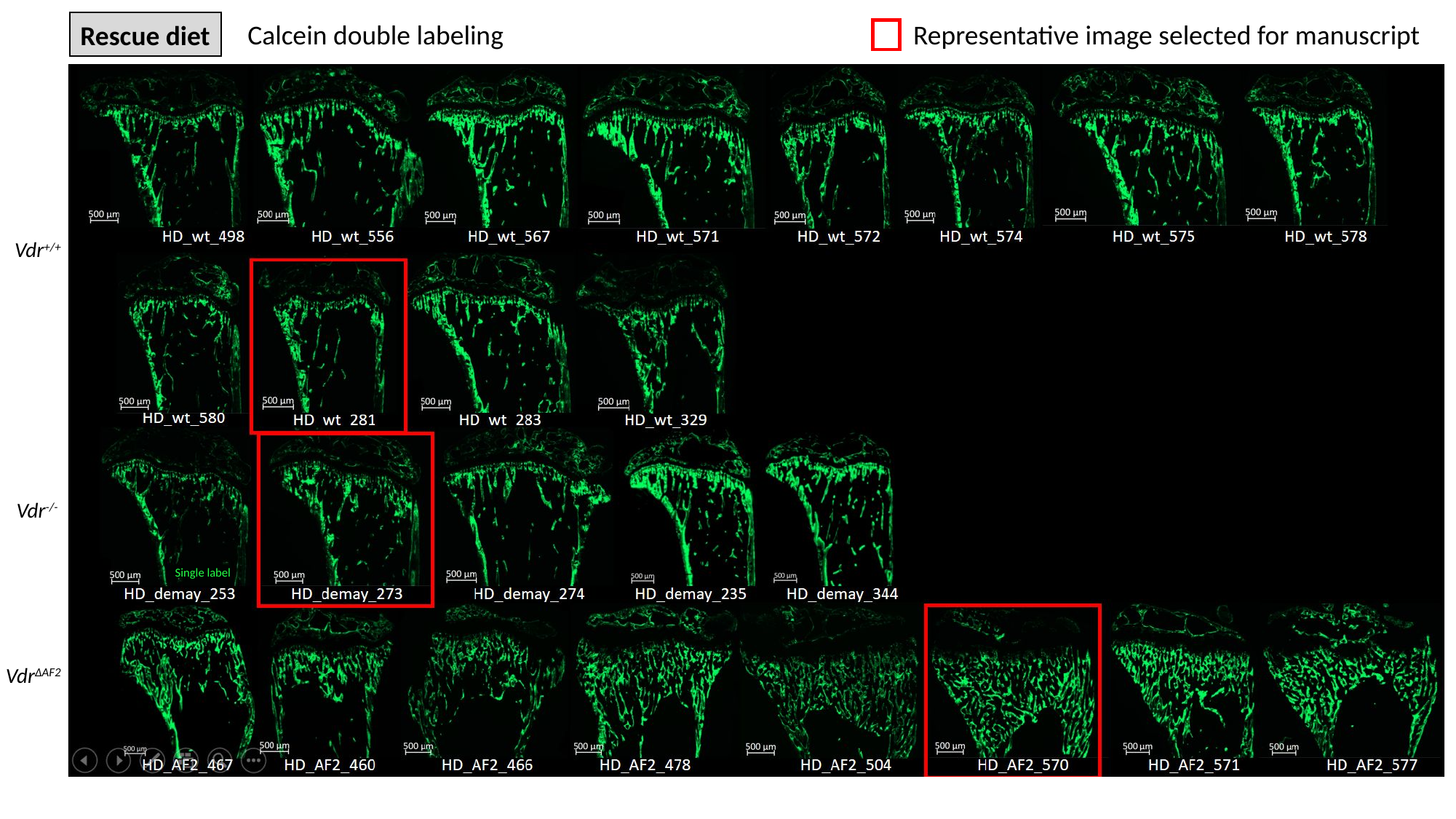

Calcein double labeling
Representative image selected for manuscript
Rescue diet
Vdr+/+
Vdr-/-
Single label
VdrΔAF2

## Slide 11
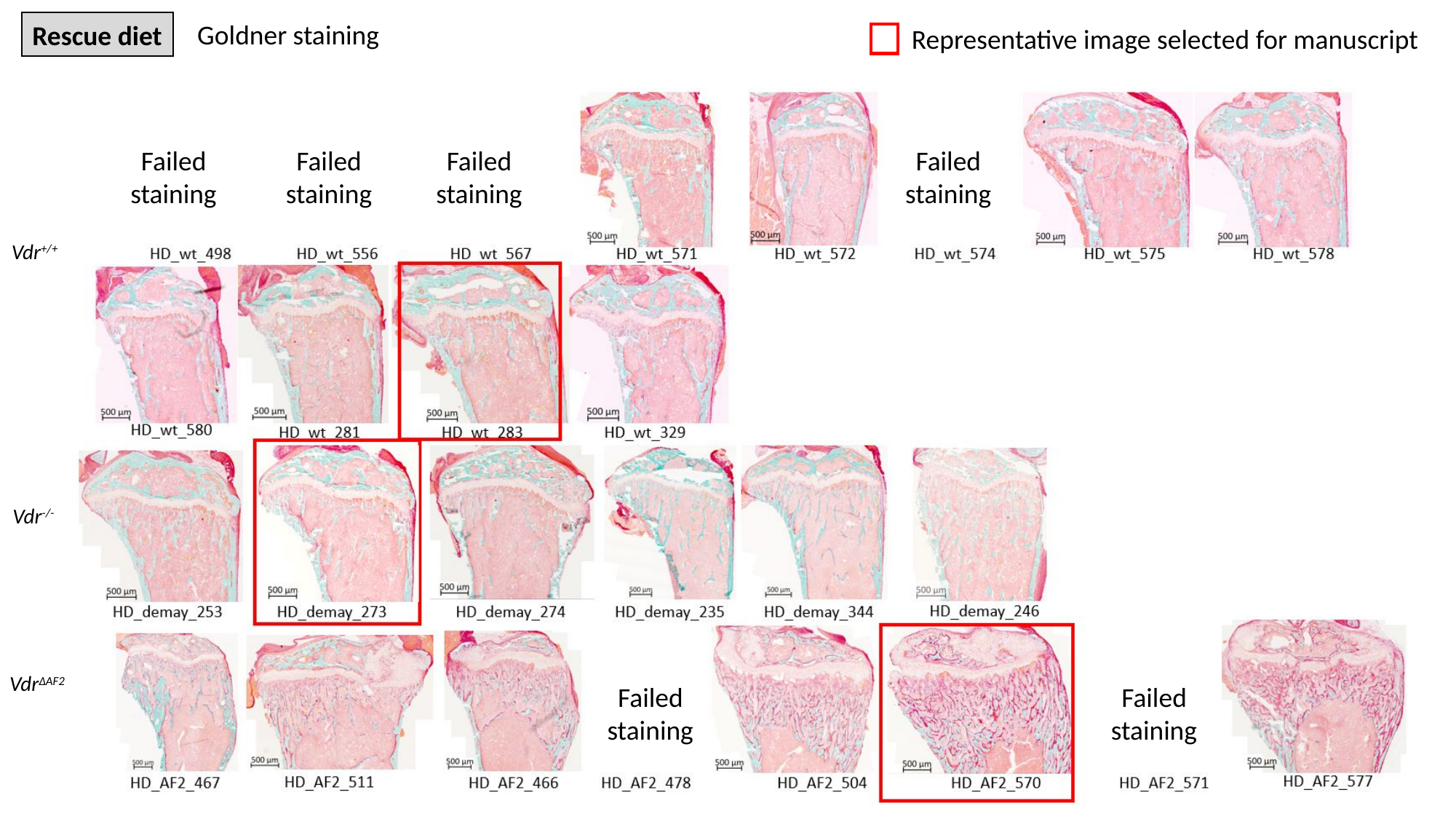

Goldner staining
Rescue diet
Representative image selected for manuscript
Failed staining
Failed staining
Failed staining
Failed staining
Failed staining
Failed staining
Vdr+/+
Vdr-/-
VdrΔAF2

## Slide 12
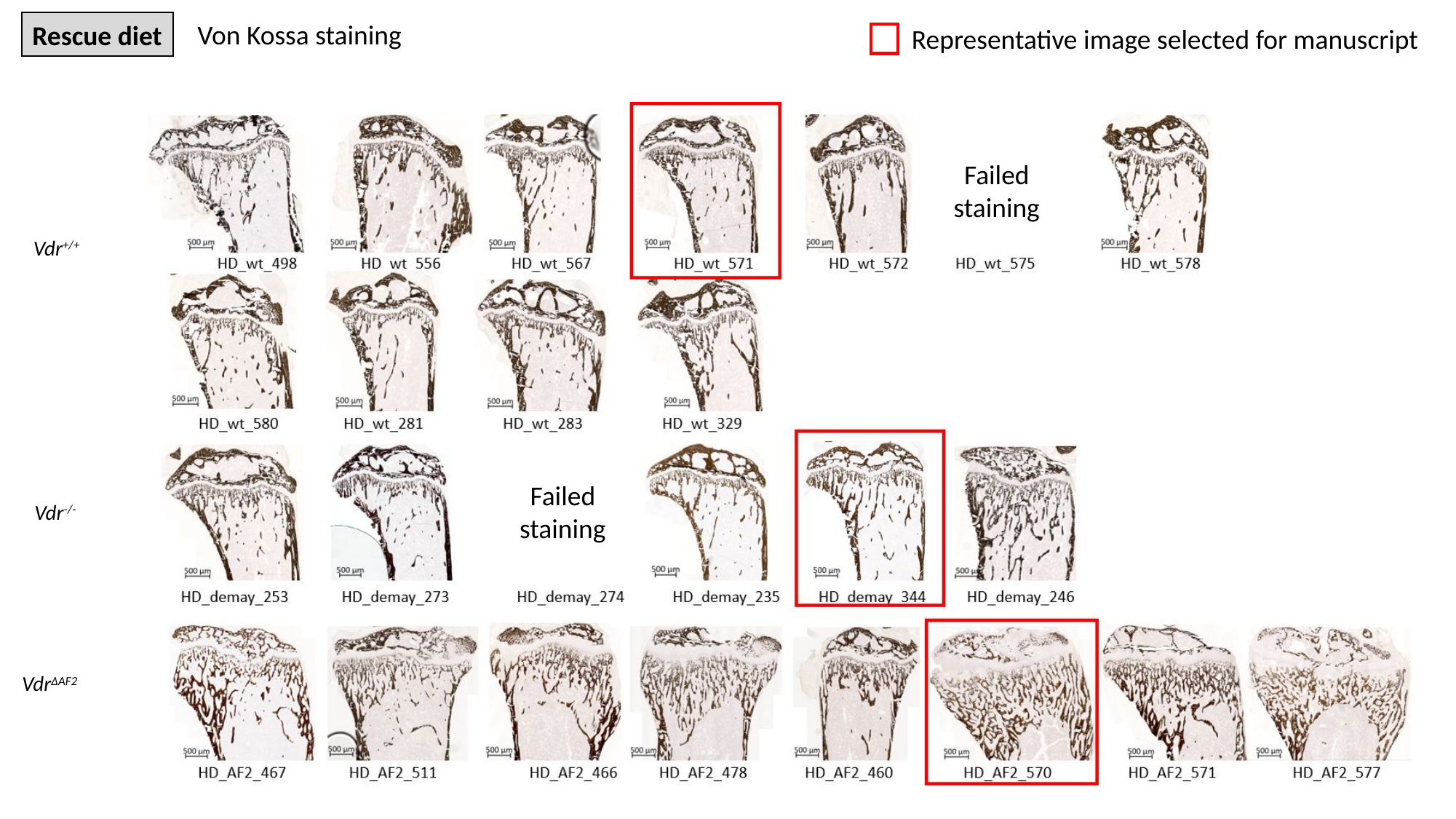

Von Kossa staining
Rescue diet
Representative image selected for manuscript
Failed staining
Failed staining
Vdr+/+
Vdr-/-
VdrΔAF2

## Slide 13
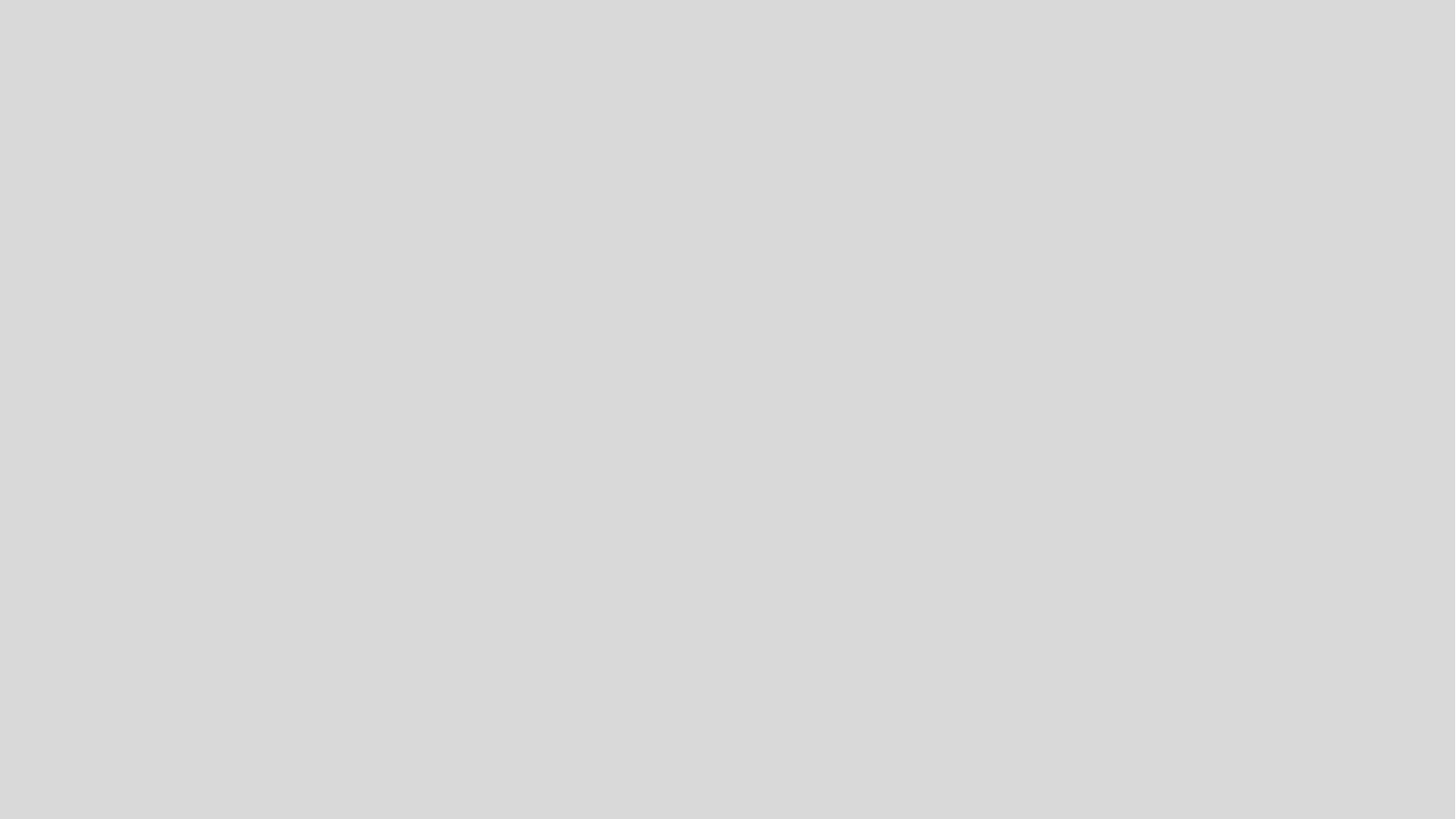

Supplement: Supplementary file 8 — Overview of bone histology and microCT [file 41413_2024_343_MOESM8_ESM.pptx]
