# Supplementary material for: Coactivator-independent vitamin D receptor signaling causes severe rickets in mice, that is not prevented by a diet high in calcium, phosphate, and lactose
Source: Bone Res. 2024 Aug 20;12:44. doi: 10.1038/s41413-024-00343-7 (PMC11335873; doi:10.1038/s41413-024-00343-7)
Supplement: Supplementary file 10 — Supplementary figure and table Legends [file 41413_2024_343_MOESM10_ESM.docx]

**Supplementary figure 1. Western blot visualizing VDR levels in *Vdr^+/+^*, *Vdr^-/-^* and *Vdr^ΔAF2^* mice. (a)** Graphic representation of VDR^+/+^, VDR^-/-^ and VDR^ΔAF2^ mutant protein structures (figure created with Biorender). **(b)** Expression of the VDR protein was examined by Western Blot analysis using 20 µg of protein lysate (n = 3) isolated from kidneys of 8-week old female *Vdr^+/+^*, *Vdr^-/-^* and *Vdr^ΔAF2^* mice maintained on the rescue diet. The primary antibody to VDR, D2K6W, was used to target an N-terminal VDR epitope, conserved in VDR^-/-^ and VDR^ΔAF2^ mutant proteins. Actin was used as a loading control.

**Supplementary figure 2. Pictures visualizing the alopecia phenotype in *Vdr^-/-^* but not in *Vdr^ΔAF2^* mice.** (**a**) Whole body pictures and (**b**) detailed pictures of the snout and chin were taken of *Vdr^+/+^*, *Vdr^-/-^* and *Vdr^ΔAF2^* mice at the indicated ages, visualizing the onset and progression of alopecia from 8 weeks onwards in *Vdr^-/-^* mice. Mice were maintained on the rescue diet.

**Supplementary figure 3. Dysregulated growth plates visualized on Safranin-O stained tibial sections.** Staining was performed on samples from 8-week-old female *Vdr^+/+^*, *Vdr^-/-^* and *Vdr^ΔAF2^* mice on normal (upper panels) or rescue diet (lower panels). Scale bars represent 500 µm.

**Supplementary figure 4. qPCR analysis of the femur of *Prrx1-Cre+/-;Vdr^lox/lox^* and *Prrx1-Cre+/-;Vdr^lox/ΔAF2^* mice.** Data expressed as Log 2 FC over average *Prrx1 Cre-* littermates.

**Supplementary figure 5.** **Calcium, phosphate and bone parameters in *LysM Cre+/- Vdr^lox/lox^* and *LysM Cre+/- Vdr^lox/ΔAF2^* mice**. **(a)** Serum calcium, phosphate, **(b)** fractional excretion of calcium and phosphate (%). **(c)** Quantification of trabecular and cortical bone parameters; BV/TV (%), trabecular (trab.) number, separation, and thickness, cortical thickness. **(e)** Absolute calcium content per femur. (**f**) Representative (based on average BV/TV %) µCT images of the sagittal plane of the proximal tibia and the transverse plane of the indicated trabecular (red squares) and cortical (blue squares) selections. (**g**) Body weight and tibia length. (**h**) Femoral qPCR analysis, data expressed as Log 2 FC over average *LysM-Cre-* littermates. All parameters were measured in 8-week-old female *LysM-Cre+/-;Vdr^lox/lox^* and *LysM-Cre+/-;Vdr^lox/ΔAF2^* mice (n = 9-15) weaned on normal diet (1 % calcium, 0.7 % phosphate).

**Supplementary table1**. µCT parameters for selecting cortical volumes of interest in relation to tibia length in tibia isolated from systemic *Vdr^+/+^*, *Vdr^-/-^* and *Vdr^ΔAF2^* mice.

**Supplementary table 2.** Genes analyzed by qPCR; primers and probe sequences.
